# Green management, access to credit, and firms’ vulnerability to the COVID-19 crisis

**DOI:** 10.1007/s11187-023-00759-1

**Published:** 2023-04-19

**Authors:** David Aristei, Manuela Gallo

**Affiliations:** Department of Economics, University of Perugia, Via Pascoli, 20, 06123 Perugia, Italy

**Keywords:** Green management, Credit constraints, Firm vulnerability, Business resilience, COVID-19 pandemic, Q50, G01, L25: G32, D22

## Abstract

This paper investigates the consequences of the COVID-19 crisis on firms’ performance and financial vulnerability. Exploiting longitudinal firm-level data from the World Bank’s “Enterprise Surveys follow-up on COVID-19” for 20 European countries, we assess whether green management quality and pre-pandemic credit access difficulties affect firms’ ability to withstand the negative impact of the pandemic. Our results indicate that green firms are more resilient to the pandemic shock. In particular, the likelihood of pandemic-induced drops in sales and liquidity significantly decreases as the quality of green management improves. Conversely, prior financing constraints strongly exacerbate the pandemic’s impact on firms’ performance and amplify liquidity stress and financing problems. Credit-constrained enterprises are not only more likely to experience liquidity shortages and repayment problems, but they also face higher difficulties in accessing bank financing. The COVID-19 crisis has also hampered the beneficial role that green management exerted on access to credit in the pre-pandemic period. During the pandemic, firms with sound environmental management practices do not benefit from improved access to finance and have a lower demand for credit, possibly suggesting a slowdown in their green investment activities.

**Supplementary information:**

The online version contains supplementary material available at 10.1007/s11187-023-00759-1.

## Abstract (plain language summary)

Green firms are more resilient to the COVID-19 shock, while prior credit access difficulties strongly exacerbate the pandemic’s impact on businesses. Using data on firms from 20 European countries, we find that enterprises with sound environmental management practices have a lower probability of pandemic-induced reductions in sales and liquidity. Pre-pandemic financing constraints significantly impair firms’ economic and financial performance and increase difficulties in accessing bank credit during the COVID-19 crisis. Our results suggest that public policies, aimed at encouraging firms’ environmentally sustainable behaviours and at supporting their access to credit, may contribute to strengthen the resilience of the economic system to unexpected shocks and favour the transition to a sustainable economy. Furthermore, the awareness of the competitive advantages offered by eco-sustainable practices may represent an incentive form firms to improve their environmental management quality and increase investment in green technologies.

**Supplementary information:**

The online version contains supplementary material available at 10.1007/s11187-023-00759-1.

## Introduction

The outbreak of the COVID-19 pandemic has abruptly and negatively affected global economic activity and triggered an exceptional crisis that greatly differs from past crises with respect to its cause, scope, and severity (Ding et al., 2021). In most countries, governments have imposed temporary lockdowns to limit the spread of the coronavirus, but these restrictions have contributed to generate enormous economic and financial distress on firms, determining a significant decline in the demand and supply of products and services and causing significant liquidity issues as a consequence of the reduction in revenues. Prolonged economic inactivity and the disruptions in supply chains and employment caused by the pandemic have strongly impaired firms’ operational and financial performance.

The dramatic impact of the COVID-19 crisis on enterprises has highlighted the need for an in‐depth analysis of the factors affecting firms’ resilience, which could provide valuable insights, both for firms and governments, on how to mitigate the adverse effects of exogenous shocks and stimulate economic recovery. Improved understanding of the elements that contribute to make firms more resilient to the pandemic shock can be useful to identify vulnerable firms and thus define targeted relief programmes, so as to lead to more effective responses to the crisis.

In light of the significant efforts made by enterprises in adapting to sustainable development goals and given their key role in fostering the transition to a green economy, it becomes crucial to assess the advantages generated by firms’ environmentally sustainable behaviours, especially in terms of ability to react to unexpected shocks. Previous literature has emphasized the relevance of green management in enhancing firms’ performance (Fernandez, 2022) and access to credit (Wellalage & Kumar, 2021). Firms with sound green management practices are considered more trustworthy by stakeholders and by financial institutions, due to their greater efficiency and profitability, better reputation, and lower environmental risk (Zhang, 2021). Only few studies have so far investigated the relationship between firms’ environmental practices and the severity of the COVID-19’s impact on business performance. Green enterprises are less prone to liquidity problems and bankruptcy during the pandemic (Wellalage & Kumar, 2020) and have better financial performance and greater resilience to the COVID-19 shock (Ding et al., 2021; Lu et al., 2022). Environmentally sustainable firms are also less likely to be severely affected by the pandemic than conventional firm (Zhang & Fang, 2022) and benefit from improved access to external finance during the COVID-19 crisis (Wellalage et al., 2022a).

Credit constraints represent a further significant factor affecting the impact of the COVID-19 outbreak on firms’ performance and financial fragility. Firms that faced credit access restrictions in the pre-pandemic period are more likely to experience liquidity problems and to be overdue on their obligations during the pandemic (Amin & Viganola, 2021; Khan, 2022). Prior external financing difficulties make enterprises less resilient to the pandemic shock and significantly more likely to experience a decline in sales (Zhang & Sogn-Grundvåg, 2022). Financial frictions exacerbate the effects of the shocks generated by the COVID-19 outbreak, by amplifying the negative effects of the pandemic on the expected sales and planned investments of credit-constrained firms (Balduzzi et al., 2020). In this respect, the heightened liquidity issues and the reduced availability of credit caused by the pandemic crisis could also limit firms’ environmental investments and trigger shifts in their green behaviours, with harmful effects on the transition to a sustainable economy (Guérin & Suntheim, 2021).

In this paper, we contribute to the growing (but still limited) literature on the economic and financial consequences of the COVID-19 crisis by analysing how green management practices, financing conditions, and other firm-level characteristics in the pre-pandemic period affect the impact of the pandemic shock on firms’ economic performance, financial fragility, and access to credit. To these aims, our empirical analysis exploits a unique longitudinal firm-level data set for 20 European countries, obtained by merging the regular World Bank’s “Enterprise Surveys” (WBES) and three rounds of the World Bank’s “Enterprise Surveys follow-up on COVID-19” (WBES-COVID). To the best of our knowledge, this is the first paper to investigate the joint role of green management quality and prior financing constraints on firms’ ability to withstand the pandemic shock by explicitly addressing potential endogeneity concerns. Our empirical strategy allows us to properly assess whether firms adopting environmental management practices are characterized by greater resilience to the pandemic shock and by lower financial fragility. Furthermore, we test whether pre-pandemic credit access difficulties contribute to exacerbate the negative impact of the COVID-19 shock on firms’ performance and amplify pandemic-related liquidity stress and financing problems. Finally, we investigate how green management quality affects firms’ access to credit during the pandemic and empirically verify whether the COVID-19 crisis has led to significant changes in demand and access to bank financing by firms engaged in green management activities.

The remainder of the paper is organized as follows. Section 2 provides an overview of the relevant literature and develops the main research hypotheses. Section 3 describes the data and defines the variables. Section 4 presents the econometric methods and discusses the main empirical findings, while Sect. 5 offers some concluding remarks.

## Literature review and hypothesis development

### Green management and firms’ resilience to the COVID-19 shock

Previous studies have shown that environmental, social, and governance (ESG) practices have beneficial effects on business performance (Aragón-Correa et al., 2008; Leonidou et al., 2016; Przychodzen & Przychodzen, 2015) and play a key role in reducing risk exposure and improving the ability to react to unexpected shocks (Bauer & Hann, 2010; de Boyrie & Pavlova, 2020; Stellner et al., 2015).

According to the *resource-based theory*, green management allows firms to develop specific capabilities that can be sources of competitive advantage (Hart, 1995; Lannelongue et al., 2017). In this respect, firms with sound environmental practices benefit from greater efficiency (Clarkson et al., 2011), cost reductions (Delmas & Pekovic, 2013), improved profitability (Shu et al., 2016), and better image and reputation (Xing et al., 2021). Fernandez (2022) finds that green management quality has a positive impact on labour productivity, overall sales, and product and process innovation. Zhang & Ma (2021) further suggest that both the number of environmental practices implemented by firms and the degree of integration of environmental management with other functions exert a positive impact on economic performance. Moreover, Soytas et al. (2019) and Gull et al. (2022) provide robust evidence supporting the positive causal effect of environmental and social sustainability initiatives on firms’ financial performance.

Sustainability performance can also improve firms’ resilience during times of crisis. From a theoretical perspective, both *stakeholder theory* and *resource-based theory* motivate the crucial role of sustainable business practices in improving firms’ ability to adjust to and recover from unexpected shocks (Huang et al., 2020; Lu et al., 2022). On the one hand, corporate sustainability fosters strong and enduring relationships with stakeholders, such as employees, suppliers, customers, and the government, which in turn can offer support and resources to adapt to and overcome the crisis. On the other hand, the competitive advantages gained through sustainable initiatives enhance organizational resilience and help firms to adapt, react, and recover from the shock. Few studies have so far investigated the role of firms’ environmentally and socially responsible behaviours and their performance during the COVID-19 crisis. Initial research has mostly focused on the association between the adoption of ESG practices and stock returns and volatility during the pandemic. Albuquerque et al. (2020) point out that the COVID-19 pandemic provides a unique opportunity to test theories of environmental and social policies and document that highly sustainable enterprises display higher returns, lower return volatility, and higher operating profit margins during the first months of pandemic. Their results also provide strong support to the relevance of customer and investor loyalty in explaining the resilience of firms with high ESG ratings. Ding et al. (2021) find that firms with stronger ESG performance experience higher stock returns during the pandemic and the sustainability–resilience nexus is stronger in countries with social norms that consider environmental and social issues as a high priority. Analogously, Broadstock et al. (2021) show that sustainability performance significantly increases stock returns and mitigates financial risk during the pandemic; they also highlight that firms with high environmental scores are more resilient and better prepared to navigate through the COVID-19 crisis. Using internationally comparable firm-level data, Zhang & Fang (2022) examine the impact of COVID-19 on business performance and provide evidence that the severity of the pandemic’s impact (measured by the time required to recover to the normal sales level) varies according to firms’ size and their environmental behaviour. In particular, they show that SMEs engaged in green management activities perform better during the pandemic than conventional SMEs and large firms. Similarly, Wellalage & Kumar (2020) suggest that enterprises adopting environmentally sustainable practices are less likely to face liquidity shortfalls and have a lower probability of insolvency or bankruptcy during the pandemic. Lu et al. (2022) also show that firms with strong sustainability performance have been more resilient to the COVID-19 shock and their financial performance has dropped less than other firms. Moreover, they suggest that mature sustainability strategies mitigate risk and provide “insurance”-like protection against economic downturns during the COVID-19 crisis.

Based on the above arguments, we posit and test our first research hypothesis:

1. Firms with better green management practices are less likely to experience a decrease in sales (HP1a) and are characterized by lower financial vulnerability (HP1b) during the COVID-19 pandemic.

### Credit constraints and firms’ vulnerabilities during the COVID-19 pandemic

Extant literature shows that financial constraints significantly hinder business performance and have strong negative effects on firms’ growth, productivity, and investment. In this respect, García-Posada Gómez (2018) points out that financial constraints have negative effects on firm investment in fixed assets, demonstrating as investments are particularly sensitive to the availability of external finance. Analogously, De Haas et al. (2020) show that constrained firms tend to reduce their investment in fixed assets and to have lower labour productivity and total sales. Berg (2018) finds that loan rejection reduces asset growth, investments, and employment, and these negative effects are concentrated among low-liquidity firms; he also demonstrates that after a loan rejection, firms cut noncash assets and increase cash holdings for precautionary savings motives, thereby amplifying the credit supply shock. Ferrando & Mulier (2022) add that not only rejected borrowers, but also discouraged borrowers (i.e. those firms that need external finance, but do not apply for a bank loan fearing rejection) have reduced investment rate and lower employment and sales growth.

Recent empirical studies have analysed the role played by credit constraints on firms’ ability to navigate through the economic disruptions caused by the pandemic. Khan (2022) shows that prior actual and perceived credit constraints exacerbate pandemic-induced credit risk as well as liquidity problems. More specifically, he points out that credit-rationed firms are more likely to experience greater liquidity problems and present a higher probability to be overdue on their obligations to financial institutions with respect to unconstrained firms. Analogously, Amin & Viganola (2021) estimate the impact of pre-pandemic credit access conditions on the likelihood of a decline in sales of the firm during the pandemic. Their results show that firms with better access to finance are significantly less likely to experience a decline in sales, particularly for firms that have a stronger and long-standing relationship with important stakeholders. Balduzzi et al. (2020) investigate the role played by credit constraints in the transmission of the shocks generated by the COVID-19 outbreak. The authors show that credit-constrained firms hold more pessimistic expectations about future sales and orders and plan to reduce employment and investment more than unconstrained firms, suggesting that financial frictions amplify the effects of the shocks generated by the COVID-19 pandemic. Fahlenbrach et al. (2021) also point out that firms with greater financial flexibility are less affected by the pandemic shock. In particular, firms that have more cash and less debt exposition before the pandemic experience a lower stock price drop than those with less financial flexibility. Zhang & Sogn-Grundvåg (2022), using firm-level survey data, assess how credit constraint conditions affect the severity of the COVID-19’s impact on firms’ performance and investigate whether firms’ obstacles in access to credit, firm size, ownership, firm age, and location can be used to identify vulnerable firms during the pandemic. Their empirical results indicate that small firms and firms with limited access to finance are more likely to aspect a longer recovery from the COVID-19 shock.

In line with previous literature, our second research hypotheses can be formulated as follows:

HP2: Prior credit constraints exacerbate the negative impact of the COVID-19 outbreak on firms’ performance (HP2a) and increase pandemic-induced financial fragility (HP2b).

### Environmental management and access to credit before and during the COVID-19 pandemic

Previous research indicates that financial institutions have incorporated environmental criteria when they make lending decisions and design loan contractual terms (Weber et al., 2015). Two main theoretical frameworks have been considered in the extant literature to explain the relationship between firms’ environmental behaviour and credit access and lending conditions: *agency theory* and *legitimacy theory*. *Agency theory* suggests that managers may hesitate to invest in green technologies, as these investments can be very expensive and they do not immediately improve the financial performance of the firm, but rather worsen of its financial condition. However, the adoption of environmentally sustainable practices boosts firms’ image and reputation, favouring their access to financial resources (Zeidan et al., 2015). Moreover, environmentally sustainable practices and compliance with environmental regulations contribute to reduce information asymmetries between firms and lending institutions, improving firms’ credit access conditions (El Ghoul et al., 2011). *Legitimacy theory* holds that firms earn environmental legitimacy when their performance with respect to the environment meets the expectations of institutions and stakeholders. Even though improving environmental performance is costly and its benefits may not necessarily be immediate, green investments can reduce unsystematic risk and enhance investors’ perception of the firm (Bansal & Clelland, 2004). Firms can further increase their environmental legitimacy by disclosing information about their own commitment to the environment. However, this may pose concerns on the value-enhancing role of green activities due to potential green-washing behaviours, since firms with poor environmental performance may use disclosure to influence public perceptions with misleading information on their actual environmental commitment (Deegan, 2002).

Empirical studies have provided significant evidence on the relationship between firms’ environmental management and access to bank financing. Zhang (2021) suggests that environmental risk affects banks’ lending decisions, due to its impact on firms’ financial performance and credit risk, and points out that environmentally sustainable firms are more likely to receive a line of credit and less likely to be imposed collateral requirements. Xing et al. (2021) show that green management significantly enhances firms’ availability of bank loans and that environmental disclosure and pressure from local governments contribute to facilitate access to credit of enterprises engaged in green management activities. Wellalage & Kumar (2021) investigate whether firms with sound environmental commitment obtain better credit access conditions and find that environmental performance has a positive impact on loan size, particularly for small and medium enterprises, while it does not impact on loan duration and collateral requirements. Accordingly, Zhang et al. (2022) show that the external assurance of environmental disclosures has a significant and positive impact on firms’ access to bank financing. Conversely, Fernandez (2022) points out that firms with above-average green management may be perceived as riskier by lending institutions and, hence, exhibit lower rates of loan approval and are subject to collateralization more frequently.

Recent literature has investigated the impact of the pandemic shock on firms’ access to credit, providing mixed evidence on how the COVID-19 crisis has affected the credit market. With respect to the demand side, as pointed out by Cowling et al. (2022a), the severe liquidity problems faced by firms since the onset of the COVID-19 crisis have led to a surge in firms’ loan demand to finance working capital needs, inducing even more reluctant discouraged borrowers to relax their distrust of banks and apply for loans. By contrast, the COVID-19 outbreak has led to a sharp contraction in business investment, due to firms’ postponement or cancellation of investment projects and negative expectations about the future, with a consequent reduction in the demand for long-term credit (Buchheim et al., 2022). Liu et al. (2022) find that the demand for credit of Chinese SMEs significantly reduced after the COVID-19 outbreak in terms of both the probability and the frequency of credit applications; such reduction was more pronounced in areas most severely affected by the pandemic and for nonstate-owned enterprises and SMEs without prior bank relationships. On the supply side, many banks changed tightened their credit standards and terms during the pandemic’s onset in response to expected higher credit losses (Bordo & Duca, 2021). However, government initiatives aimed at strengthening banks’ lending capacity and at incentivizing their lending activities have played a key role in sustaining the flow of credit to firms during the pandemic (Casanova et al., 2021; Cascarino et al., 2022). In this respect, Marsh & Sharma (2021) show that public credit guarantees offset a potentially large credit crunch in the USA by stimulating growth in loans covered by the guarantee and by preventing declines in non-guaranteed loans. Accordingly, Calabrese et al. (2022) and Cowling et al. (2022b) demonstrate that the COVID-19 loan guarantee schemes implemented in the UK have been effective for supporting small business during the pandemic and for protecting them from future loan default.

The effects of the pandemic shock on the credit market may have also affected the relevance of environmental factors in banks’ lending decisions. To date, only few empirical studies have examined the role of firms’ environmental behaviour on access to bank credit during the COVID-19 pandemic. Wellalage & Kumar (2020) show that the green firms have easier access to credit during the pandemic. Firms with greater environmental performance create value and cooperation from stakeholders that leads to an increase in access to bank financing. Furthermore, Wellalage et al. (2022a) investigate the association between firm-level environmental performance and firm financing during the pandemic, providing evidence on the degree to which environmental performance is advantageous when the market experiences an adverse shock. The authors find a significant positive relationship between environmental performance and access to external financing, demonstrating that a firm’s engagement in environmental activities provides several benefits in a crisis period, as it creates a higher level of trust between firms and shareholders and contributes to increase stakeholder collaboration.

Based on these insights, we posit and test the following hypothesis:

HP3: Green management quality contributes to enhance firms’ access to credit not only in normal market conditions, but also during the COVID-19 crisis.

## Data and measurement

### Data sources

The empirical analyses carried out in this paper rely on two main data sources: World Bank’s “Enterprise Surveys” (WBES) and the World Bank’s “Enterprise Surveys follow-up on COVID-19” (WBES-COVID). The WBES provides detailed and comparable information on firms’ ownership and governance, performance, export and innovation activities, and financing conditions, based on nationally representative stratified samples of enterprises operating in manufacturing, retail, and service sectors. To the aims of our analysis, we focus on firms from 20 European countries (Albania, Bulgaria, Croatia, Cyprus, Czech Republic, Estonia, Georgia, Greece, Hungary, Italy, Latvia, Lithuania, Malta, Moldova, North Macedonia, Poland, Portugal, Romania, Slovak Republic, and Slovenia) and consider data from the latest releases of the WBES before the COVID-19 pandemic, which were conducted in 2019 in all the countries considered with the exception of Greece, where it was carried out in 2018.

During the pandemic, firms interviewed in the regular WBES were re-contacted with the aim of assessing the economic consequences of COVID-19 on businesses. In particular, the WBES-COVID provides timely and detailed information on changes in firms’ sales, employment, and liquidity after the COVID-19 outbreak, on financial vulnerabilities and access to credit, on how firms have coped with the pandemic, and on their expectations on the recovery from the shock. In all the countries considered (with the exception of Albania), three rounds of the WBES-COVID were conducted via phone interviews over the period between May 2020 and January 2022, with an average response rate of about 85% in the countries considered.^1^ To the aims of our analysis, we merge the regular WBES and the three rounds of the WBES-COVID and obtain a unique longitudinal data set that allows us to investigate how green management practices, financing constraints, and other firm-level characteristics in the pre-pandemic period have affected the impact of the COVID-19 shock on firms’ sales, liquidity, financial fragility, and access to credit. Our main estimation sample consists of 9519 enterprises, which participated to at least one of the WBES-COVID rounds and for which we have complete information on pre-pandemic characteristics, for a total of 23,147 firm-round observations. Appendix Table 10 reports the sample distribution by country and industrial sector.

### The economic and financial consequences of COVID-19 on firms

We exploit data from the WBES-COVID to investigate the consequences of the COVID-19 pandemic on firms’ performance and financial vulnerability. To this aim, we first focus on the impact on sales and, as in Amin & Viganola (2021), define a binary indicator equal to one for those firms reporting a decline in sales with respect to the same month of the previous year (*Sales decreased*) and zero otherwise. To further assess the impact on the level of sales during the pandemic, we consider the percentage change in sales since the same month of the previous year (*Sales change*). We also analyse firms’ expectations on the time necessary to recover from the pandemic shock and define an ordinal variable (*Expected recovery*) that categorize the expected number of months needed for sales to get back to normal into 4 categories (i.e. “Current sales are as normal”; “1 to 6 months”; “7 to 12 months”; “More than 12 months/never”).

We then focus on different dimensions of firm-level financial vulnerability induced by the COVID-19 pandemic. We first analyse the probability of experiencing pandemic-induced liquidity problems and define a binary indicator (*Liquidity decreased*) identifying firms that have reported a shortfall in liquidity or cash flow since the outbreak of COVID-19 (or since the previous round of the WBES-COVID for firms interviewed in more than one round). Moreover, we consider firms’ repayment difficulties and define two binary indicators identifying firms that have delayed payments to suppliers, landlords, or tax authorities for more than one week (*Delayed Payments*) and that have been overdue on obligations to any financial institution (*Financial obligations overdue*) since the outbreak of COVID-19 (or since the previous round for firms interviewed in multiple rounds).

Complete variable definitions are reported in Appendix Table 11, while Table 1 reports descriptive statistics on the economic and financial consequences of COVID-19 disaggregated by country. From Table 1, we notice the dramatic consequences of the COVID-19 crisis on firms’ operational and financial performance. More than 53% of firms experienced a decrease in sales during the pandemic, and the level of sales decreased on average by about 16.5 percentage points. Furthermore, less than 49% of the enterprises in the sample reported that their sales are back to normal pre-pandemic levels, while more than 11% of businesses expect to recover from COVID-19 crisis in more than 12 months or never. With respect to pandemic-induced firm-level financial fragility, we observe that 47.5% of the firms experienced decreased liquidity or cash flow availability since the COVID-19 outbreak. The pandemic has also strongly undermined firms’ repayment ability: more than 28% of businesses have delayed payments for more than 1 week, and about 10% have been overdue on their obligations to financial institutions during since the onset of the COVID-19 crisis.

**Table 1:** The consequences of the COVID-19 shock on firms

Country	Sales decreased	Sales change	Current sales as normal	Recover in more than 12 months/never	Liquidity decreased	Delayed payments	Financial obligations overdue
Albania	0.8797	− 50.01	0.1527	0.0220	0.7265	0.6847	0.2065
Bulgaria	0.6136	− 18.01	0.4257	0.0718	0.6296	0.2293	0.0361
Croatia	0.4250	− 9.81	0.5800	0.1346	0.3978	0.2440	0.0678
Cyprus	0.5439	− 19.52	0.5600	0.0820	0.4746	0.2630	0.0814
Czech Republic	0.4800	− 13.88	0.1693	0.0876	0.4486	0.2361	0.0484
Estonia	0.3741	− 5.98	0.3606	0.0844	0.3374	0.2660	0.0211
Georgia	0.5561	− 25.32	0.6440	0.1160	0.5731	0.2708	0.0874
Greece	0.6838	− 24.14	0.4711	0.1250	0.6517	0.3141	0.1045
Hungary	0.4044	− 9.48	0.5859	0.0633	0.3682	0.0864	0.0146
Italy	0.5825	− 23.73	0.5212	0.0604	0.5754	0.4064	0.0764
Latvia	0.4230	− 13.47	0.4923	0.0633	0.4145	0.2499	0.1205
Lithuania	0.5189	− 12.53	0.4413	0.0744	0.4937	0.3237	0.0953
Malta	0.5276	− 13.94	0.3493	0.0810	0.4957	0.1954	0.0270
Moldova	0.7077	− 33.42	0.3894	0.0709	0.6887	0.4702	0.1681
North Macedonia	0.5811	− 16.32	0.4566	0.0449	0.5423	0.3787	0.1365
Poland	0.5218	− 14.16	0.2125	0.1588	0.4291	0.2538	0.1289
Portugal	0.5161	− 15.89	0.4991	0.0366	0.4688	0.1861	0.0392
Romania	0.4713	− 10.68	0.3536	0.1015	0.4403	0.2643	0.0502
Slovak Republic	0.5128	− 15.05	0.3313	0.1040	0.4730	0.2625	0.0145
Slovenia	0.4469	− 7.08	0.2415	0.1154	0.4651	0.2877	0.0827
Total	0.5343	− 16.40	0.4861	0.1151	0.4748	0.2812	0.1014

Proportions and average values are computed using sample weights, rescaled by the inverse of the number of observations in each country

### Measuring green management

One of the main aims of our analysis is to assess whether firms adopting green management practices before the outbreak of COVID-19 are less vulnerable to the pandemic shock. For this reason, we exploit the detailed information on environmental management activities provided in the “Green Economy Module” of the regular WBES to define a green management score. This module asks questions about four main types of green management practices: strategic objectives related to the environment and climate change (1 question), managers’ environmental responsibility (3 questions), monitoring and auditing of emissions and usage of resources (9 questions), and environmental targets (3 questions).^2^ Based on firms’ replies to these questions, we follow the approach proposed by De Haas et al., (2020, 2022) to construct an overall environmental management score. To this aim, we first normalize the scores for each question so that they have zero mean and unit standard deviation. We then compute an unweighted average of these *z*-scores for each of the four areas of green management. Lastly, we create an overall green management *z*-score as an unweighted average of the scores for the four areas and standardize this unweighted average once more. This overall score accounts for the different green management practices implemented by firms, including the definition of green policies and strategies and the use of management assessment tools, as well as actions to achieve compliance with environmental laws and regulations. Coherently with Zhang & Ma (2021), it also allows controlling for the complexity of a firm’s green management and its integration with other managerial processes and functions, like strategic planning, production operations, and quality management. Table 2 reports the average values of the green management score in each of the countries considered, while Supplementary Appendix Fig. S1 shows country-level distribution of the score.

**Table 2:** Firms’ green management and access to credit

	(a) Before the COVID-19 pandemic				(b) During the COVID-19 pandemic	
Country	Green management	Financing constraints	Applied credit	Credit denied	Applied credit	Credit denied
Albania	− 0.1958	0.0471	0.1653	0.0000	–	–
Bulgaria	0.0293	0.1912	0.1412	0.0216	0.0569	0.1836
Croatia	0.0024	0.0238	0.2323	0.0424	0.0994	0.2400
Cyprus	0.0883	0.0905	0.1398	0.1618	0.1159	0.1474
Czech Republic	0.2726	0.0344	0.2692	0.0094	0.0923	0.1746
Estonia	0.0937	0.0354	0.2435	0.0992	0.1250	0.1301
Georgia	− 0.3633	0.1174	0.3220	0.1154	0.2240	0.0077
Greece	0.3392	0.2147	0.1105	0.1529	0.2128	0.2421
Hungary	0.0588	0.0671	0.2551	0.0112	0.0971	0.0569
Italy	0.0092	0.1322	0.0965	0.0830	0.3082	0.0359
Latvia	0.3426	0.0674	0.2304	0.0271	0.1697	0.3758
Lithuania	− 0.0797	0.1111	0.2154	0.1480	0.0801	0.1445
Malta	− 0.0092	0.0224	0.2001	0.0920	0.1228	0.0000
Moldova	− 0.1527	0.3482	0.1970	0.3560	0.2121	0.3012
North Macedonia	0.2690	0.1477	0.1705	0.0720	0.3296	0.1520
Poland	− 0.1513	0.1128	0.1235	0.0148	0.1812	0.1697
Portugal	− 0.2066	0.0576	0.1558	0.1186	0.1807	0.0517
Romania	− 0.1071	0.2922	0.1365	0.1768	0.1625	0.1270
Slovak Republic	0.1338	0.0990	0.1569	0.0364	0.0872	0.1318
Slovenia	0.2344	0.0260	0.3199	0.0521	0.2294	0.1246
Total	0.0000	0.1162	0.1417	0.0462	0.1897	0.1333

Proportions and average values are computed using sample weights, rescaled by the inverse of the number of observations in each country

### Firms’ access to credit

In our empirical analysis, we also aim at investigating whether credit constraints before the COVID-19 outbreak represent a main determinant of firms’ economic and financial fragility during the pandemic. We thus exploit the detailed loan application data from the pre-pandemic WBES and construct a direct survey-based proxy for difficulties in accessing bank credit, which encompasses both formal rejection by banks and self-rationing by firms (i.e. credit discouragement). Specifically, we define a binary variable (*Credit constraints*) which identifies as credit-constrained those firms that, in the last regular WBES before to the pandemic, reported that they applied for a line of credit or a bank loan and were denied credit or that they did not apply due to unfavourable credit conditions and/or fear of rejection. Thus, as in García-Posada Gómez (2018) and De Haas et al. (2022), we consider as non-financially constrained those firms that either had no need for additional credit or whose demand for credit was satisfied.

Furthermore, we also investigate the role of green management and other pre-pandemic firm-level characteristics on access to bank credit and financing difficulties during the COVID-19 crisis. For this reason, using the information on loan application provided in rounds 2 and 3 of the WBES-COVID, we first define a binary indicator of credit demand (*Applied Credit*) equal to one if the firm applied for a line of credit or a bank loan since the outbreak of COVID-19 (or since round 2 of the WBES-COVID, for firms re-interviewed in round 3) and zero otherwise. Conditional on credit demand, we then construct a binary indicator of credit rationing (*Credit denied*) that equals one if the firm’s most recent application for a line of credit or a bank loan during the COVID-19 pandemic was rejected (and zero if it was approved).

Table 2 reports the average values of all the credit access indicators for each country, before and during the COVID-19 crisis. We notice that firms’ demand for additional bank financing has significantly increased during the pandemic with respect to the pre-crisis period, due to the heightened cash flow pressures experienced by businesses. At the same time, access to credit has strongly worsened since the COVID-19 outbreak. In particular, the percentage of firms having faced a rejection of their loan or credit line applications has increased from the 4.6% in the pre-pandemic period to more than 13%, suggesting that, despite the loan guarantee schemes implemented by governments to support firms’ access to credit, banks have not lowered their lending standards during the pandemic.

### Control variables

To properly assess the role of green management and credit constraints and mitigate as much as possible omitted variable bias, we control for a large set of pre-pandemic firm-level characteristics, exploiting the detailed information provided by the regular WBES.

As in previous empirical studies (e.g. Khan, 2022; Zhang & Fang, 2022), we control for firm age and number of employees (both included in logarithmic terms to account for possible non-linear effects) and to variables related to its ownership structure and legal status (*Sole proprietorship*, *Domestic* and *Family owned*). As in Birindelli et al. (2022) and Wellalage et al. (2022b), we also assess whether female-managed firms are more vulnerable to the COVID-19 shock than their male counterparts and define an indicator of female involvement in the management of the firm (*Female managed*) that equals one when the firm’s top manager is a woman. We account for business opacity and quality disclosure by means of binary indicators for having financial statements certified by an external auditor (*Audited*) and for having obtained an internationally recognized quality certification (*Quality certification*). We further control for managerial experience, proxied by the logarithm of years of experience in the sector of the top manager (*Manager experience*), and control for export activity (*Direct exporter*). A binary variable identifying firms located in cities with more than one million inhabitants (*Large city*) is also included to account for the environment in which the firm operates.

As in Fernandez (2022) and Zhang & Sogn-Grundvåg (2022), we control for the number of products and services contracted with banks (*Financial openness*) and include a dummy for the use of informal sources (i.e. moneylenders, friends, and relatives) to finance the purchase of fixed assets or working capital (*Informal financing*) and a dummy identifying those firms that use internal funds or retained earnings as the main source of financing for their working capital needs (*Internal financing*).

Using the information provided in the WBES-COVID, we also control for the number of weeks that the firm was temporarily closed due to the COVID-19 outbreak (*Weeks closed*). This allows us to account for the pandemic-related impact on firms’ operations, which significantly varies across countries, sectors, and time, depending on the stages of the pandemic and on the measures that national governments adopted to limit the spread of the coronavirus. Given the significant variation in the timing of the survey rounds, we also include the number of days between the day of interview and 11 March 2020, the day in which the World Health Organization (WHO) declared COVID-19 as a pandemic (*Days since pandemic*). Moreover, we control for the impact of government policies and interventions to contain the spreading of the coronavirus and consider the *Stringency index* developed by the Oxford COVID-19 Government Response Tracker (OxCGRT) (Hale et al., 2021). This index is composed of nine individual country-level indicators of government containment measures (school closures, workplace closures, cancellation of public events, restrictions on gatherings, public transport closures, stay-at-home requirements, restrictions on internal movements, international travel bans, and public information campaigns) rescaled to range between 0 and 100, with higher values indicating stricter restrictions. Information on the *Stringency index* from the OxCGRT are matched with firm-level data from the WBES-COVID based on the firm’s country of location and day of interview.^3^ Finally, we account for country- and sector-level heterogeneity by means of country and industry fixed effects and include survey round dummies.

Appendix Table 11 provides complete definitions and descriptive statistics for all the explanatory variables considered in the analysis, while Supplementary Appendix Table S3 reports the pairwise correlation matrix.

## Empirical analysis

### The impact of COVID-19 on firms’ performance

In this sub-section, we assess how green management practices and pre-pandemic financing difficulties affect the impact of the COVID-19 outbreak on firms’ performance. We first focus on the probability of having experienced a pandemic-induced drop in sales and specify the following panel probit model:

1 $$\begin{array}{c}{Sales\;decreased}_{it}=1(\gamma{Green\;management}_{i0}+\delta{Credit\;constraints}_{i0}\\+{{\varvec{x}}}_{i0}\boldsymbol'{\boldsymbol\beta}_1+{\lambda_1Weeks\;closed}_{it}+{\lambda_2Days\;since\;pandemic}_{id}\\+{\lambda_3Stringency\;index}_{cd}+\theta_{1c}+\theta_{1s}+\theta_t+a_i+\varepsilon_{it}>0)\end{array}$$

where $1\left(\cdot \right)$ is an indicator function equal to one if the expression in parentheses is true and zero otherwise; ${{\varvec{x}}}_{it0}$ is a vector of exogenous pre-pandemic firm characteristics; ${\theta }_{1c}$, ${\theta }_{1s}$, and ${\theta }_{t}$ are country, sector, and survey wave fixed effects; ${a}_{i}$ is time-constant unobserved heterogeneity; and ${\varepsilon }_{it}$ is a standard normal idiosyncratic error, capturing unobserved time-varying factors, independent of the regressors conditional on ${a}_{i}$.

A potential issue in model (1) is that both firms’ environmental behaviour and their credit access conditions may be endogenous with respect to the economic consequences of the COVID-19 outbreak. Firms’ environmental behaviour, driven by both externally imposed legal requirements and proactive environmental strategies, may be endogenously determined with their performance and thus with their ability to react to unexpected shocks. As discussed in Garcia-Castro et al. (2010) and Soytas et al. (2019), unobservable firm-specific characteristics, such as internal decision-making style, quality of top management, unobserved productivity, and managers’ ethical attitudes, may affect both firms’ decisions to engage in green management practices and their economic and financial performance and in turn their resilience to the pandemic shock (Zhang & Fang, 2022). At the same time, as pointed out by Khan (2022) and Zhang & Sogn-Grundvåg (2022), the likelihood of facing credit access difficulties and the resilience to the pandemic shock may be jointly affected by unobservable factors related to a firm’s quality, creditworthiness, and ability to adjust its business model, giving rise to endogeneity issues. For these reasons, we extend model (1) to allow ${\varepsilon }_{it}$ to be correlated with both ${Green\; management}_{i0}$ and ${Credit\; constraint}_{i0}$ and specify a reduced form linear equation for ${Green\; management}_{i0}$:

2 $${Green\; management}_{i0}={{\varvec{x}}}_{i0}\boldsymbol'{{\varvec{\beta}}}_{2}{+{\varvec{z}}}_{i01}\boldsymbol'{\boldsymbol{\alpha }}_{1}+{\theta }_{2c}+{\theta }_{2s}+{u}_{i0}$$

and a reduced form probit equation for ${Credit\; constraint}_{i0}$:

3 $${Credit\; constraints}_{i0}=1\left({{\varvec{x}}}_{i0}\boldsymbol'{{\varvec{\beta}}}_{3}{+{\varvec{z}}}_{i02}\boldsymbol'{\boldsymbol{\alpha }}_{2}+{\theta }_{3c}+{\theta }_{3s}+{v}_{i0}>0\right)$$

with normal idiosyncratic error terms ${u}_{i0}$ and ${v}_{i0}$, independent of the regressors and potentially correlated with ${\varepsilon }_{it}$, and where the vectors ${{\varvec{z}}}_{i01}$ and ${{\varvec{z}}}_{i02}$ includes strictly exogenous regressors excluded from (1). In this respect, we consider firms’ perception of business licencing, trade regulations, corruption, tax rates, and administration as obstacles to its operations (*Business obstacles*) as an additional instrumental variable in both Eqs. (2) and (3), assuming that perceived obstacles directly affect a firm’s environmental management and credit access but do not exert a direct impact on businesses’ performance and vulnerability during the COVID-19 pandemic. Furthermore, as in Wellalage & Kumar (2021), we use the green management score of nearby firms (i.e. those operating in the same region of the firm) (*Peer green management*) as an instrument for a firm’s green management practices. This choice rests on the assumption that firms, operating in regions where the average quality of green management is higher, have a better knowledge of green practices and tend to have higher environmental management scores. Similarly, following De Haas et al. (2022), we use the proportion of nearby firms, excluding those operating in the same sector, that experienced credit access difficulties before the pandemic (*Peer credit constraints*) as an instrument for a firm’s financing constraints. Since shocks in a firm’s access to credit do not affect the financing conditions of the other firms operating in the same region, but in a different sector, this variable can be safely considered as an exogenous indicator of local financing conditions in the pre-pandemic period.

Parameters of the recursive system composed of Eqs. (1), (2), and (3) can be estimated jointly by means of maximum likelihood estimation (MLE). Here, we adopt a pooled (i.e. partial) MLE method that, despite being inefficient compared to a random-effects approach, does not restrict error dependence over time and is robust to serial correlation and to any distributional misspecification other than the conditional mean. As discussed in Wooldridge (2010), pooled MLE represents a convenient and computationally simple estimation method in non-linear panel data models. The only additional complication is that a robust variance matrix estimator is needed to account for serial correlation: clustering at the individual-level allows obtaining valid standard errors and test statistics.

Column 1 of Table 3 reports the average marginal effects (AMEs) estimated from endogenous probit model for the probability of having experienced a decrease in sales during the pandemic. Before discussing the estimated AMEs, we first assess the validity of our identification strategy and test the exogeneity of firms’ green management and financing constraints. Results of the *F* tests for the joint significance of the instrumental variables in the reduced form equations allow us to reject the null hypothesis that the instruments are weak at the 1% level, supporting the assumptions underlying our identification strategy. In particular, marginal effects estimated from reduced form models (reported in Supplementary Appendix Table S4) suggest that perceived business obstacles significantly increase both the environmental management score and the probability of facing credit constraints; furthermore, coherently with De Haas et al. (2022), we find that a firm’s green management score increases with the quality of green management of the other firms operating in the same region and that firms are more likely to be credit constrained if nearby enterprises operating in different sectors are also constrained. After having provided support for the instruments’ validity, we assess the endogeneity of green management and financing constraints by testing the significance of the correlation between ${\varepsilon }_{it}$ and the error terms ${u}_{i0}$ and ${v}_{i0}$. Results from these formal exogeneity tests indicate that both the regressors cannot be considered as exogenously determined with respect to the probability of a decrease in sales during the COVID-19 pandemic (*p* values equal to 0.0061 and 0.0022). This evidence suggests that the endogenous probit model should be preferred against the standard probit, as it allows us to address the endogeneity of both green management and credit constraints and obtain consistent parameter estimates.

**Table 3:** Firms’ sales during the COVID-19 pandemic: estimated AMEs

Model	Pooled endogenous probit	Pooled endogenous linear regression
Dependent variable	Sales decreased	Sales change
	(1)	(2)
Green management	−0.0652***	3.6130**
	(0.0216)	(1.4732)
Credit constraints	0.2179***	− 7.8772***
	(0.0584)	(1.8803)
Age (in logs)	0.0107**	0.0820
	(0.0052)	(0.4199)
Employees (in logs)	− 0.0012	0.9327***
	(0.0056)	(0.3372)
Sole proprietorship	0.0063	− 0.1711
	(0.0112)	(0.6786)
Domestic	0.0215	− 0.8429
	(0.0136)	(0.8783)
Female-led	− 0.0037	− 0.3821
	(0.0093)	(0.5880)
Family owned	0.0132*	− 0.4926
	(0.0079)	(0.4969)
Audited	0.0109	− 1.0187
	(0.0100)	(0.6349)
Quality certification	− 0.0019	0.5847
	(0.0110)	(0.6844)
Manager experience	0.0034	− 0.3886
	(0.0060)	(0.3975)
Direct exporter	0.0044	− 1.1855*
	(0.0101)	(0.6316)
Trade credit	− 0.0023	0.6155
	(0.0086)	(0.5291)
Financial openness	0.0159**	− 0.2837
	(0.0073)	(0.4376)
Informal financing	0.0239	− 3.6599***
	(0.0203)	(1.2386)
Internal financing	0.0240***	− 1.7388***
	(0.0086)	(0.5201)
Large city	0.0330***	− 1.4474**
	(0.0101)	(0.6813)
Weeks closed	0.0084***	− 0.8319***
	(0.0006)	(0.0473)
Days since pandemic	− 0.0005***	0.0782***
	(0.0001)	(0.0089)
Stringency index	0.0016***	− 0.1678***
	(0.0003)	(0.0225)
Country and sector FEs	Yes	Yes
Survey round FEs	Yes	Yes
Weak-instrument *F* test		
* Green management*	[0.0000]	[0.0000]
* Credit constraints*	[0.0000]	[0.0000]
Wald test of exogeneity		
* Credit constraints*	[0.0061]	[0.0297]
* Green management*	[0.0022]	[0.0082]
Observations	23,147	22,945

The table reports the average marginal effects on the probability of a decrease in sales and on the percentage change in sales compared with the same month of the previous year, estimated from an endogenous probit model and an endogenous linear regression, respectively. Standard errors, clustered at the firm level, are reported below the estimates. We use *Business Obstacles*, *Peer green management*, and *Peer credit constraints* as additional instruments for the endogenous regressors *Green management* and *Credit constraints*. The *p* values of the *F* tests for weak instruments and of the Wald tests of exogeneity are reported in square brackets

^***^, **, and * denote significance at the 1, 5, and 10% levels, respectively

Turning to the analysis of the estimated AMEs, we provide strong empirical evidence on the beneficial role of environmental management on firms’ performance during the COVID-19 crisis. In line with the findings of Zhang & Fang (2022), firms adopting green management practices are less likely to have experienced a decrease in sales since the outbreak of the pandemic, providing support to the validity of our research hypothesis HP1a. Specifically, a one standard deviation increase in the green management score reduces the probability of a decrease in sales by about 6.5 percentage points. Moreover, we find that having faced credit access difficulties before the pandemic significantly increases the probability of a shortfall in sales during the COVID-19 crisis by about 21.8 percentage points. This evidence suggests that prior credit constraints strongly exacerbate the negative impact of the pandemic on firms’ performance, confirming the findings of recent empirical studies (Khan, 2022; Zhang & Sogn-Grundvåg, 2022) and supporting our research hypothesis HP2a.^4^

With respect to control variables, our empirical results indicate that older and family-owned firms, as well as those mainly relying on internal funds for their working capital needs, have a higher probability of a sales shortfall during the pandemic. Firms operating in large cities are more likely to be affected by the COVID-19 outbreak, due to the more severe impact of the pandemic and of the related prevention measures in larger and densely populated urban areas. In this respect, the probability of a decrease in sales significantly increases with the duration of temporary closures due to COVID-19-related restrictions: an additional week of temporary closure increases the probability of a sales decrease by 0.84 percentage points. We also show that the likelihood of experiencing a decrease in sales significantly lowers with the number of days since the pandemic began. This result suggests that over the course of the pandemic, enterprises have become better able to weather the crisis and recover from the initial negative shock to sales. Moreover, we find that a 1% increase in the *Stringency index* raises the probability of a shortfall in firms’ sales by 0.16 percentage points, suggesting that the stringency of country-level government initiatives to limit the spreading of the coronavirus significantly contributes to impair firms’ operations.^5^

We deepen the analysis on the impact of COVID-19 crisis on firms’ economic performance by analysing the annual percentage chance in sales during the pandemic. To this aim, we specify the following linear panel data model:

4 $$\begin{array}{c}{Sales\;change}_{it}=\gamma{Green\;management}_{i0}+\delta{Credit\;constraints}_{i0}\\+{{\varvec{x}}}_{i0}\boldsymbol'{\boldsymbol\beta}_1+{\lambda_1Weeks\;closed}_{it}+{\lambda_2Days\;since\;pandemic}_{id}\\+{\lambda_3Stringency\;index}_{cd}+\theta_{1c}+\theta_{1s}+\theta_t+a_i+\varepsilon_{it}>0\end{array}$$

The endogeneity of green management and financing constraints is again taken into account by estimating Eq. (4) jointly with the reduced form Eqs. (2) and (3) by pooled MLE. Estimation results are presented in column 2 of Table 3. Also in this case, we find that the instruments considered are valid and that both green management and credit constraints are endogenously determined with respect to the percentage decrease in sales during the COVID-19 pandemic.

From the estimated AMEs, we notice that a one standard deviation increase in the green management score raises sales by more than 3.6 percentage points. This evidence confirms that environmental management quality significantly enhances firms’ resilience to the COVID-19 shock. Conversely, pre-pandemic credit constraints are found to amplify the adverse effects of the pandemic, in line with the findings of Amin & Viganola (2021). Specifically, firms that have experienced financing difficulties before the pandemic are characterized by a variation in sales with respect to the previous year that is 7.9 percentage points lower than those with no credit access issues.

We also find that smaller enterprises and firms that directly export their products or services, recur to informal financing, use internal funds to finance their day-to-day operations, and are located in large cities are characterized by a significantly lower sales growth. Moreover, temporary shutdowns are found to strongly impact on the level of sales, with a 1-week increase in the duration of temporary closures reducing sales growth by 0.83 percentage points. Also in this case, we find that sales growth increases with the number of days since the onset of the pandemic, suggesting that firms are moving towards a recovery from the dramatic impact of COVID-19 outbreak on their sales. Coherently with the findings of Janzen & Radulescu (2022), firms’ operational performance is found to be strongly hampered by the stringency of country-level containment policies, with a 1% increase in the *Stringency index* leading to a decrease of firms’ sales by about 0.17 percentage points.

We further investigate whether environmental management and prior financing difficulties affect the severity of the pandemic impact, measured in terms of firms’ expectations on the length of recovery from the COVID-19 shock. We thus specify an ordered probit model for the ordinal dependent variable *Expected recovery* as follows:

5 $$\begin{array}{c}{Expected\;recovery}_{it}=h\;\mathrm{if}\;\kappa_{h-1}<(\gamma{Green\;management}_{i0}\\+\delta{Credit\;constraints}_{i0}+{{\varvec{x}}}_{i0}\boldsymbol'{\boldsymbol\beta}_1+{\lambda_1Weeks\;closed}_{it}\\+{\lambda_2Days\;since\;pandemic}_{id}+{\lambda_3Stringency\;index}_{cd}\\+\theta_{1c}+\theta_{1s}+\theta_t+a_i+\varepsilon_{it})\leq\kappa_h\end{array}$$

where ${\kappa }_{h}$ ($h=0,\dots ,3$) are threshold parameters and ${\varepsilon }_{it}$ is a standard normal idiosyncratic error. Endogeneity issues, due to unobserved factors that may affect both the expected severity of the COVID-19 shock and firms’ environmental behaviour and credit constraints, are taken into account by specifying Eq. (5) together with the reduced forms (2) and (3) and jointly estimating parameters by pooled MLE methods.

Table 4 reports the average marginal effects on the probability of each ordered outcome. Before commenting the estimated AMEs, from the lower part of the table, we notice that the instruments considered are relevant, supporting the identifying assumptions underlying our instrumentation strategy. Furthermore, the null hypothesis of exogeneity is rejected for both green management and credit constraints, supporting the appropriateness of an endogenous ordered probit model.

**Table 4:** Severity of COVID-19 impact on firms: estimated AMEs

Model	Pooled endogenous ordered probit			
Dependent variable	Expected recovery			
Ordered outcome	Current sales as normal	1 to 6 months	7 to 12 months	More than 12 months/never
	(1)	(2)	(3)	(4)
Green management	0.0468**	− 0.0111**	− 0.0175**	− 0.0181**
	(0.0210)	(0.0047)	(0.0077)	(0.0086)
Credit constraints	− 0.2808***	0.0666***	0.1054***	0.1088***
	(0.0626)	(0.0140)	(0.0214)	(0.0276)
Age	− 0.0009	0.0002	0.0003	0.0004
	(0.0060)	(0.0014)	(0.0023)	(0.0023)
Employees	0.0050	− 0.0012	− 0.0019	− 0.0019
	(0.0055)	(0.0013)	(0.0021)	(0.0021)
Sole proprietorship	− 0.0085	0.0020	0.0032	0.0033
	(0.0116)	(0.0027)	(0.0043)	(0.0045)
Domestic	− 0.0197	0.0047	0.0074	0.0076
	(0.0134)	(0.0032)	(0.0051)	(0.0051)
Female-led	− 0.0074	0.0018	0.0028	0.0029
	(0.0094)	(0.0022)	(0.0035)	(0.0036)
Family owned	− 0.0193**	0.0046**	0.0072**	0.0075**
	(0.0082)	(0.0020)	(0.0031)	(0.0032)
Audited	− 0.0095	0.0023	0.0036	0.0037
	(0.0099)	(0.0023)	(0.0037)	(0.0039)
Quality certification	0.0060	− 0.0014	− 0.0022	− 0.0023
	(0.0108)	(0.0026)	(0.0041)	(0.0042)
Manager experience	− 0.0019	0.0005	0.0007	0.0007
	(0.0058)	(0.0014)	(0.0022)	(0.0022)
Direct exporter	− 0.0037	0.0009	0.0014	0.0014
	(0.0100)	(0.0024)	(0.0038)	(0.0039)
Trade credit	− 0.0026	0.0006	0.0010	0.0010
	(0.0087)	(0.0021)	(0.0033)	(0.0034)
Financial openness	− 0.0085	0.0020	0.0032	0.0033
	(0.0074)	(0.0017)	(0.0028)	(0.0029)
Informal financing	− 0.0038	0.0009	0.0014	0.0015
	(0.0199)	(0.0047)	(0.0075)	(0.0077)
Internal financing	− 0.0150*	0.0036*	0.0056*	0.0058*
	(0.0087)	(0.0021)	(0.0033)	(0.0034)
Large city	− 0.0406***	0.0096***	0.0152***	0.0157***
	(0.0102)	(0.0025)	(0.0039)	(0.0040)
Weeks closed	− 0.0097***	0.0023***	0.0036***	0.0038***
	(0.0005)	(0.0002)	(0.0003)	(0.0002)
Days since pandemic	0.0004***	− 0.0001***	− 0.0001***	− 0.0001***
	(0.0001)	(0.0000)	(0.0000)	(0.0000)
Stringency index	− 0.0004	0.0001	0.0002	0.0002
	(0.0003)	(0.0001)	(0.0001)	(0.0001)
Country and sector FEs	Yes			
Survey round FEs	Yes			
Weak-instrument *F* test				
* Green management*	[0.0000]			
* Credit constraints*	[0.0000]			
Wald test of exogeneity				
* Green management*	[0.0189]			
* Credit constraints*	[0.0011]			
Observations	20,468			

The table reports the average marginal effects on the probability of each level of the *Expected recovery* variable, estimated from a pooled endogenous ordered probit model. Standard errors, clustered at the firm level, are reported below the estimates. We use *Business Obstacles*, *Peer green management*, and *Peer credit constraints* as additional instruments for the endogenous regressors *Green management* and *Credit constraints*. The *p* values of the *F* tests for weak instruments and of the Wald tests of exogeneity are reported in square brackets

^***^, **, and * denote significance at the 1, 5, and 10% levels, respectively

Analysing the estimated AMEs, we point out that environmental management quality significantly improves firms’ resilience to the pandemic shock, coherently with the finding of Zhang and Fang (2022). Specifically, a one standard error increase in the green management score raises the probability that a firm’s current sales are as normal by about 4.7 percentage points, while it reduces by about 1.8 percentage points the probability of a moderate (7–12 months) and a severe (more than 12 months/never) impact of the COVID-19 crisis in terms of expected months for recovery.

Firms that faced financing difficulties before the pandemic’s onset are instead 28.1% less likely to have returned to their normal level of sales at the time of the interview. Moreover, perceptions on the time necessary to recover from the pandemic shock are much more pessimistic for credit-constrained enterprises, which are 10.5 and 10.9% more likely to recover in 7–12 months and in more than 1 year (or never), respectively, compared to unconstrained firms. In line with Zhang & Sogn-Grundvåg (2022), this evidence confirms that pre-pandemic credit access difficulties strongly hamper firms’ ability to withstand the pandemic impact and slow down their recovery.

We do not find significant differences in the expected severity of the COVID-19’s impact based on most of the firm-level characteristics considered in the analysis: only family-owned firms, those that do not recur to external financing for their working capital needs, and those located in large cities are less likely to have returned to their normal sales level and tend to foresee a longer recovery. Moreover, we again point out that firms that have been temporarily closed for a longer period are more likely to experience a lengthy recovery: an additional week of temporary closure leads to a 0.97% decrease in the probability of having returned to normal sales at the time of interview and raises the probability of a prolonged recovery by about 0.4 percentage points. We also notice that the expected recovery time significantly decreases with the number of days since the COVID-19 outbreak, confirming that firms’ ability to manage the challenges caused by the COVID-19 pandemic has improved over time. Conversely, firms’ expectations on the recovery from the pandemic shock are not affected by the stringency of the government initiatives taken at the country level to contain the virus.

### Green management, credit constraints, and financial fragility during the COVID-19 pandemic

In this section, we assess the role of green management and prior credit access difficulties in affecting firms’ financial fragility during the pandemic. To this aim, we address the potential endogeneity of both environmental behaviour and credit constraints with respect to the probability of experiencing liquidity shortfalls and financial problems, by estimating the following panel probit model jointly with the reduced form Eqs. (2) and (3):

6 $$\begin{array}{c}{Financial\;fragility}_{it}=1(\gamma{Green\;management}_{i0}+\delta{Credit\;conststraints}_{i0}\\+{{\varvec{x}}}_{i0}\boldsymbol'{\boldsymbol\beta}_1+\lambda_1{Weeks\;closed}_{it}+{\lambda_2Days\;since\;pandemic}_{id}\\+{\lambda_3Stringency\;index}_{cd}+\theta_{1c}+\theta_{1s}+\theta_t+a_i+\varepsilon_{it}>0)\end{array}$$

where ${Financial fragility}_{it}$ represents the alternative binary indicators of financial difficulties experienced during the COVID-19 pandemic considered in the analysis (i.e. *Liquidity decreased*, *Delayed payments*, and *Financial obligations overdue*).

Estimation results are presented in Table 5. Results from weak instrument tests confirm the validity of our identification strategy. Exogeneity tests indicate that the environmental management score and the credit constraints indicator are endogenous determinants of the probability of a liquidity shortfall, while only the financing constraints indicator is endogenously determined with respect to the probability of being overdue on financial obligations. Both the variables can be instead considered as exogenous with respect to the probability of having delayed payments.

**Table 5:** Firms’ financial fragility since the COVID-19 outbreak: estimated AMEs

Model	Pooled endogenous probit	Pooled endogenous probit	Pooled endogenous probit
Dependent variable	Liquidity decreased	Delayed payments	Financial obligations overdue
	(1)	(2)	(3)
Green management Credit constraints	− 0.0678***	0.0003	− 0.0014
	(0.0223)	(0.0038)	(0.0021)
	0.1888***	0.0514***	0.0701***
	(0.0494)	(0.0111)	(0.0216)
Age	0.0022	− 0.0156***	− 0.0072**
	(0.0061)	(0.0057)	(0.0030)
Employees	− 0.0082	− 0.0118***	− 0.0047**
	(0.0057)	(0.0035)	(0.0020)
Sole proprietorship	0.0146	0.0008	− 0.0004
	(0.0112)	(0.0108)	(0.0055)
Domestic	0.0312**	0.0438***	0.0172**
	(0.0137)	(0.0127)	(0.0072)
Female-led	0.0134*	0.0094	0.0080*
	(0.0080)	(0.0088)	(0.0047)
Family owned	0.0171**	0.0074	− 0.0017
	(0.0080)	(0.0077)	(0.0041)
Audited	0.0010	− 0.0076	0.0027
	(0.0101)	(0.0083)	(0.0046)
Quality certification	− 0.0088	− 0.0279***	− 0.0082*
	(0.0113)	(0.0087)	(0.0050)
Manager experience	− 0.0044	− 0.0187***	0.0004
	(0.0059)	(0.0056)	(0.0029)
Direct exporter	0.0075	− 0.0076	− 0.0017
	(0.0101)	(0.0088)	(0.0049)
Trade credit	0.0004	0.0316***	− 0.0035
	(0.0085)	(0.0082)	(0.0045)
Financial openness	0.0089	− 0.0066	0.0013
	(0.0072)	(0.0066)	(0.0037)
Informal financing	0.0410**	0.0246	0.0022
	(0.0199)	(0.0178)	(0.0094)
Internal financing	0.0053	− 0.0315***	− 0.0162***
	(0.0085)	(0.0079)	(0.0043)
Large city	0.0228**	0.0339***	− 0.0008
	(0.0100)	(0.0095)	(0.0053)
Weeks closed	0.0115***	0.0050***	0.0018***
	(0.0008)	(0.0005)	(0.0002)
Days since pandemic	0.0000	− 0.0001	0.0001*
	(0.0001)	(0.0001)	(0.0001)
Stringency index	0.0011***	− 0.0004	− 0.0001
	(0.0003)	(0.0003)	(0.0002)
Country and sector FEs	Yes	Yes	Yes
Survey round FEs	Yes	Yes	Yes
Weak-instrument *F* test			
* Green management*	[0.0000]	[0.0000]	[0.0000]
* Credit constraints*	[0.0000]	[0.0000]	[0.0000]
Wald test of exogeneity			
* Green management*	[0.0030]	[0.2487]	[0.1705]
* Credit constraints*	[0.0077]	[0.3468]	[0.0198]
Observations	23,147	21,431	22,320

The table reports the average marginal effects on the probability of having experienced a decrease in liquidity, delays in payments, and financial obligations overdue, estimated from endogenous probit models. Standard errors, clustered at the firm level, are reported below the estimates. We use *Business Obstacles*, *Peer green management*, and *Peer credit constraints* as additional instruments for the endogenous regressors *Green management* and *Credit constraints*. The *p* values of the *F* tests for weak instruments and of the Wald tests of exogeneity are reported in square brackets

^***^, **, and * denote significance at the 1, 5, and 10% levels, respectively

Focusing on the probability of a shortfall in liquidity or cash flow (column 1 of Table 5), the estimated average marginal effects show that firms with better environmental management are significantly less likely to have experienced liquidity issues during the COVID-19 crisis, coherently with the findings of Wellalage & Kumar (2020) and with our research hypothesis HP1b. We find that a one standard deviation increase in the green management score reduces the probability of liquidity shortfall by 6.8 percentage points, confirming that green management quality strongly improves firms’ financial soundness and contributes to alleviate the liquidity issues triggered by the pandemic shock.

The estimated partial effect for *Credit constraints* shows that firms that experienced financing constraints before the COVID-19 outbreak are, *ceteris paribus*, 18.9% more likely to experience liquidity and cash flow problems than unconstrained firms. This result is consistent with our predictions (HP2b) and with the findings of Khan (2022) and confirms that firms with prior credit access difficulties are less able to cope with the cash flow shocks induced by the COVID-19 outbreak.

Additionally, the pandemic triggers liquidity issues more often for domestic, family-owned, and female-led enterprises, as well as for firms resorting to informal financing sources and for those located in large cities. At the same time, firms that were temporarily closed for longer periods are significantly more likely to face pandemic-induced liquidity stress. Each additional week of temporary closure increases the probability of a decrease in liquidity by about 1.2 percentage points, as the consequent disruption in business revenue streams drains liquidity for all kinds of firms. We also find that the probability of a decrease in liquidity is not significantly affected by the number of days since the pandemic’s outbreak, suggesting that the cash flow issues caused by the COVID-19 crisis tend to persist over time. Finally, we provide evidence on the detrimental impact of country-level containment measures on firms’ liquidity: a 1% increases in the *Stringency index* significantly raises the likelihood of a cash flow shortfall by about 0.11 percentage points.

In column 2 and 3 of Table 5, we report the results for the probabilities of having delayed payments and of being overdue on financial obligations, as proxies for a firm’s repayment difficulties and credit risk. The estimated AMEs show that financially constrained firms are about 5.1 and 7.0% more likely than unconstrained firms to have delayed payments and to be overdue on their financial obligations since the onset of the pandemic, respectively. This evidence is in line with the results of Khan (2022) and provides support to our predictions (HP2b), further emphasizing the detrimental effect of prior credit access problems on firms’ financial fragility. Focusing on green management, we find that neither the probability of facing repayment difficulties during the pandemic nor the probability of being overdue on financial obligations are significantly affected by a firm’s green management quality.

With respect to the other firm-level control variables, we find similar determinants of late payments and arrears in financial obligations. Younger, smaller, and domestic firms, as well as those lacking internationally recognized quality certifications, have a higher probability of delaying payments and being overdue in their financial obligations. Similarly, enterprises that mainly rely on internally generated funds to finance their working capital are significantly less likely to experience repayment difficulties and arrears during the pandemic, as they have higher internal liquidity to cope with their payment obligations and are less dependent on external financing. We also find that female-led enterprises have a higher probability to be overdue in meeting their obligations to financial institutions than their male counterparts during the COVID-19 crisis. Moreover, firms resorting to trade credit before the COVID-19 outbreak are significantly more likely to experience pandemic-induced repayment difficulties, as well as those whose top manager has a lower experience in the sector and those located in large cities. We further show that the probabilities of late payments and financial obligations overdue significantly increase with the number of weeks the firm was temporarily closed, while the overall stringency of country-level containment policies does not significantly affect firms’ repayment difficulties. Finally, we find that repayment difficulties do not significantly lessen and tend to persist over time, while the probability of being overdue on financial obligations increases with the number of days since the onset of the pandemic. This latter evidence may be indicative of the excessive firms’ indebtedness and increased solvency problems emerging from the COVID-19 crisis.

### Robustness and additional analyses

In this section, we conduct a number of additional analyses to assess the robustness of our main findings on the role of green management and credit constraints in shaping firms’ resilience to the pandemic shock.

First, in order to control for changes in the sample composition over time, we re-estimated all the empirical models of Sects. 4.1 and 4.2 on the balanced panel composed of the 5709 firms for which we have complete data for all the three rounds of the WBES-COVID (which represent about 60% of the firms in the sample). The estimated marginal effects presented in Table 6 are substantially similar to those obtained in the unbalanced sample, providing support to the robustness of our main empirical findings to the time dimension of the panel and suggesting that the effects of green management quality and financial constraints on firms’ resilience remain significant over the whole period of analysis.

**Table 6:** Robustness: estimation on the balanced panel

	Panel A: firms’ sales during the COVID-19 pandemic				
Dependent variable	Sales decreased	Sales change			
Green management	− 0.0733***	3.6166**			
	(0.0260)	(1.8046)			
Credit constraints	0.2383***	− 8.8894***			
	(0.0716)	(2.1079)			
Observations	17,127	17,127			
	Panel B: severity of COVID-19 impact on firms				
Dependent variable	Expected recovery				
Ordered outcome	Current sales as normal	1 to 6 months		7 to 12 months	More than 12 months/never
Green management	0.0533**	− 0.0127**		− 0.0194**	− 0.0211**
	(0.0252)	(0.0057)		(0.0090)	(0.0116)
Credit constraints	− 0.3022***	0.0722***		0.1103***	0.1196***
	(0.0650)	(0.0147)		(0.0212)	(0.0297)
Observations	17,127				
	Panel C: firms’ financial fragility since the COVID-19 outbreak				
Dependent variable	Liquidity decreased	Delayed payments		Financial obligations overdue	
Green management	− 0.0658***	0.0007		− 0.0001	
	(0.0211)	(0.0045)		(0.0025)	
Credit constraints	0.1798***	0.0522***		0.0894***	
	(0.0637)	(0.0145)		(0.0270)	
Observations	17,127	17,127		17,127	

The table reports the average marginal effects obtained from the regression models presented in Sects. 4.1 and 4.2 estimated on the balanced panel composed of the 5709 firms for which we have complete data for all the three rounds of the WBES-COVID. Standard errors, clustered at the firm level, are reported below the estimates. All the regressions include the controls used in Tables 3, 4, and 5. Complete estimation results are available upon request

^***^, **, and * denote significance at the 1, 5, and 10% levels, respectively

We further assess whether the effect of green management quality on the ability to withstand the COVID-19 crisis differs between rationed and non-rationed enterprises. To this aim, we extend the baseline models to include the interaction term ${Green\; management}_{i0}\times {Credit\; constraints}_{i0}$, and, following Ai & Norton (2003), we compute the interaction effect as the discrete difference, with respect to the dummy *Credit constraints*, of the partial derivative of the outcome of interest with respect to the continuous *Green management z*-score. Table 7 presents the estimated average marginal effects of *Green management* and *Credit constraints*, together with the corresponding average interaction effects. None of the interaction effects is statistically significant, suggesting that the impact of green management quality on firms’ performance and financial fragility during the pandemic does not change depending on the firm’s pre-pandemic credit access conditions.^6^

**Table 7:** Robustness: testing for interaction effects

	Panel A: firms’ sales during the COVID-19 pandemic				
Dependent variable	Sales decreased	Sales change			
Green management	− 0.0646***	3.5866**			
	(0.0217)	(1.4725)			
Credit constraints	0.2128***	− 7.8363***			
	(0.0552)	(1.8977)			
Interaction effect	− 0.0010	0.3016			
	(0.0119)	(0.7741)			
Observations	23,147	22,945			
	Panel B: severity of COVID-19 impact on firms				
Dependent variable	Expected recovery				
Ordered outcome	Current sales as normal	1 to 6 months		7 to 12 months	More than 12 months/never
Green management	0.0468**	− 0.0111**		− 0.0176**	− 0.0181**
	(0.0211)	(0.0048)		(0.0077)	(0.0086)
Credit constraints	− 0.2700***	0.0652***		0.1006***	0.1541***
	(0.0552)	(0.0142)		(0.0164)	(0.0276)
Interaction effect	− 0.0075	0.0042		0.0048	− 0.0194
	(0.0109)	(0.0034)		(0.0052)	(0.0121)
Observations	20,468				
	Panel C: firms’ financial fragility since the COVID-19 outbreak				
Dependent variable	Liquidity decreased	Delayed payments		Financial obligations overdue	
Green management	− 0.0668***	0.0002		− 0.0014	
	(0.0223)	(0.0038)		(0.0021)	
Credit constraints	0.1883***	0.0545***		0.0701***	
	(0.0493)	(0.0121)		(0.0216)	
Interaction effect	− 0.0099	0.0136		− 0.0011	
	(0.0114)	(0.0122)		(0.0017)	
Observations	23,147	21,431		22,320	

The table reports the average marginal effects estimated from the regression models presented in Sects. 4.1 and 4.2, extended to include an interaction term between the continuous *Green management z*-score and the binary indicator *Credit constraints*. Standard errors, clustered at the firm level, are reported below the estimates. All the regressions include the controls used in Tables 3, 4, and 5. Complete estimation results are available upon request

^***^, **, and * denote significance at the 1, 5, and 10% levels, respectively

Finally, as the beneficial effect of green management on firms’ performance during the pandemic may only reflect the role of good management practices, we extend our baseline models to control for the quality of overall management. To this aim, we exploit the information on management practices based on eleven questions from the “Management practices” section of the WBES, which are however asked only to firms with at least 20 employees (which represent about the 55% of the firms in the sample). These questions cover four main areas of management practices: operations (1 question), monitoring (2 questions), targeting (4 questions), and incentives (4 questions). As we have previously done for the *Green Management* score, following Bloom & Van Reenen (2007) and Bloom et al. (2012), we define a *General Management* score by standardizing the scores for each question, averaging them for each individual area, computing an unweighted average of the scores for the four areas and standardize it once more.^7^ We first re-estimated all the baseline models on the sub-sample of firms with at least 20 employees to assess whether our main empirical findings are confirmed when micro and small enterprises are excluded from the estimation sample. Results are reported in Table 8 and largely confirm that green management significantly improves firms’ resilience to the pandemic shock, while credit constraints strongly amplify the negative impact of the COVID-19 outbreak. However, it is worth remarking that the magnitude of the marginal effects is lower compared to those obtained in the entire sample. We then extend all the empirical models to include the *General Management z*-score and, as in De Haas et al. (2022), control for its potential endogeneity, using the general management of nearby enterprises (i.e. operating in the same region of the firm) as additional instrumental variable. Results in Table 8 confirm that pre-pandemic credit access difficulties exacerbate the adverse impact of the COVID-19 crisis on firms’ performance and financial fragility. Furthermore, we find that firms’ resilience to the pandemic shock significantly increases with both green and general management scores. Firms with better overall management practices are less likely to experience pandemic-induced drops in sales, have lower liquidity issues, and are less likely to face repayment difficulties, coherently with the findings of Grover and Karplus (2021). At the same time, the beneficial role of green management quality in protecting firms from sales and liquidity shortfalls remains confirmed, with estimated marginal effects larger than those of the overall management one. Overall, the evidence obtained highlights that green and general management are two distinct drivers of firms’ resilience. These findings also suggest that our baseline models allow us capturing the actual role of green management quality in mitigating the pandemic’s impact on firms and not simply the effect of good general management practices.

**Table 8:** Robustness: controlling for general management quality

	Panel A: firms’ sales during the COVID-19 pandemic							
Dependent variable	Sales decreased		Sales change					
Green management	− 0.0320***	− 0.0308***	3.0242**	2.7568**				
	(0.0050)	(0.0056)	(1.5049)	(1.1381)				
Credit constraints	0.0948***	0.0883***	− 7.0324***	− 6.7168***				
	(0.0167)	(0.0168)	(2.0887)	(1.9846)				
General management		− 0.0107*		1.3322**				
		(0.0059)		(0.5586)				
Observations	12,504		12,389					
	Panel B: severity of COVID-19 impact on firms							
Dependent variable	Expected recovery							
Ordered outcome	Current sales as normal		1 to 6 months		7 to 12 months		More than 12 months/never	
Green management	0.0457***	0.0514***	− 0.0127***	− 0.0141***	− 0.0177***	− 0.0199***	− 0.0153***	− 0.0173***
	(0.0141)	(0.0152)	(0.0036)	(0.0051)	(0.0049)	(0.0051)	(0.0048)	(0.0059)
Credit constraints	− 0.2282***	− 0.2148***	0.0632***	0.0591***	0.0885***	0.0833***	0.0765***	0.0724***
	(0.0510)	(0.0566)	(0.0162)	(0.0148)	(0.0138)	(0.0165)	(0.0238)	(0.0261)
General management		0.0177**		− 0.0049**		− 0.0069**		− 0.0060**
		(0.0088)		(0.0025)		(0.0032)		(0.0028)
Observations	11,185							
	Panel C: firms’ financial fragility since the COVID-19 outbreak							
Dependent variable	Liquidity decreased		Delayed payments		Financial obligations overdue			
Green management	− 0.0499***	− 0.0506***	− 0.0049	− 0.0021	− 0.0025	− 0.0014		
	(0.0148)	(0.0154)	(0.0044)	(0.0050)	(0.0024)	(0.0027)		
Credit constraints	0.1423***	0.1348***	0.0503***	0.0491***	0.0305***	0.0318***		
	(0.0367)	(0.0369)	(0.0152)	(0.0153)	(0.0075)	(0.0075)		
General management		− 0.0198**		− 0.0150***		− 0.0058**		
		(0.0757)		(0.0052)		(0.0027)		
Observations	12,504		11,540		12,055			

The table reports the average marginal effects obtained from the regression models presented in Sects. 4.1 and 4.2, estimated on the sub-sample of firms with at least 20 employees and extended to include the *General management z*-score. Standard errors, clustered at the firm level, are reported below the estimates. All the regressions include the controls used in Tables 3, 4, and 5. Complete estimation results are available upon request

^***^, **, and * denote significance at the 1, 5, and 10% levels, respectively

### Firms’ green management and access to credit during the COVID-19 pandemic

Finally, we assess the role of green management on firms’ access to credit during the COVID-19 pandemic. We focus on the probability of credit denial and specify a panel probit model with endogenous sample selection, to account for the selection bias that may arise as firms that are more likely to have an application rejected may be also more likely to refrain from applying (Brown et al., 2011). Formally:

7 $$\begin{array}{l}Credit\;denied_{it}=1(\gamma_1Green\;management_{\;io}\;+\delta_1\;Credit\;constraints_{i\mathit0}\\\qquad\qquad\qquad\quad+\boldsymbol x_{i0}\boldsymbol '{\boldsymbol\beta}_1+\lambda_{11}\;Weeks\;closed_{it}+\lambda_{21}\;Days\;since\;pandemic_{id}\\\qquad\qquad\qquad\quad+{\lambda_{31}Stringency\;index}_{cd}+\theta_{1c}+\theta_{1s}+\theta_{1t}+a_{1i}+\varepsilon_{it}>0)\\\qquad\qquad\qquad\quad\qquad\qquad{if\;Apply\;credit}_{it}=1\\{Apply\;credit}_{it}=1({\gamma_2Green\;management}\;_{i0}+\delta_2{Credit\;constraints}_{i0}\\\qquad\qquad\qquad\quad+\boldsymbol x_{i0}^{}\boldsymbol'{\boldsymbol\beta}_2+{\lambda_{12}Weeks\;closed}_{it}+{\lambda_{22}Days\mathit\;since\mathit\;pandemic}_{id}\\\qquad\qquad\qquad\quad+{\lambda_{32}Stringency\;index}_{cd}+{\alpha_1Internal\;financing}_{i0}\\\qquad\qquad\qquad\quad+{\alpha_2Business\;Obstacles}_{i0}+\theta_{2c}+\theta_{2s}+\theta_{2t}+a_{2i}+\omega_{it}>0)\end{array}$$

where the outcome variable ${Credit\; denied}_{it}$ is observed only when ${Apply\; credit}_{it}=1$ (selection mechanism) and the errors ${\varepsilon }_{it}$ and ${\omega }_{it}$ follow a bivariate standard normal distribution with arbitrary correlation ${\rho }_{\varepsilon \omega }$. Endogenous selectivity operates through errors correlation: when ${\rho }_{\varepsilon \omega }\ne 0$ a simple univariate probit model for ${Credit\; denied}_{it}$ on the selected sample leads to inconsistent estimates. To improve parameter identifiability, we follow Brown et al. (2011) and exclude the *Business obstacles* and *Internal financing* binary indicators from the outcome equation for ${Credit denied}_{it}$, assuming that a firm’s perception of its business environment and its access to internal funding directly affect its loan demand behaviour, but not the bank’s actual loan granting decision.

Model (7) is further extended to accommodate for the potential endogeneity of environmental management and pre-pandemic credit access difficulties with respect to firms’ credit demand behaviour and credit rejection probability during the COVID-19 crisis. As discussed in the extant literature (Fernandez, 2022; Wellalage & Kumar, 2021; Xing et al., 2020), the endogeneity of green management may be due to the fact that firms’ environmental behaviour and financing policies may be jointly determined by unobservable factors related to firm quality, attitudes towards risk, and creditworthiness. At the same time, previous studies (Aristei & Angori, 2022; Pigini et al., 2016) have shown that credit demand and financing constraints tend to persist over time due to persistence in observed and unobserved heterogeneity and to true state dependence, highlighting the necessity of accounting for the endogeneity of pre-pandemic financing conditions. Moreover, Cowling et al. (2022a) recently show that firms that have been credit rejected in the pre-pandemic period have a lower loan demand during the COVID-19 crisis and that enterprises scarred by previous rejection are significantly less likely to be granted credit during the pandemic.

Table 9 reports the average marginal effects on the conditional probability of credit rejection and on the probability of having applied for a line of credit or a bank loan during the pandemic, estimated from the endogenous probit model with sample selection. Before discussing the estimated AMEs, from the lower part of the table, we firstly notice that the correlation coefficient ${\rho }_{\varepsilon \omega }$ between the error terms of credit demand and rationing equations is statistically significant at the 1% level, confirming the necessity of accounting for endogenous sample selection.^8^ Results of the weak instrument and exogeneity tests provide support to the validity of our identification strategy and indicate that firms’ environmental management practices and pre-pandemic credit access difficulties are endogenously determined with respect to both credit demand and rationing probabilities during the COVID-19 crisis.

**Table 9:** Firms’ access to credit during and before the COVID-19 pandemic: estimated AMEs

	(a) During the COVID-19 pandemic		(b) Before the COVID-19 pandemic	
Dependent variable	Credit denied	Applied credit	Credit denied	Applied credit
	(1)	(2)	(3)	(4)
Green management	0.0304	− 0.0520**	− 0.1196***	0.0868**
	(0.0405)	(0.0221)	(0.0458)	(0.0399)
Credit constraints	0.1149**	− 0.1040***		
	(0.0508)	(0.0094)		
Age	0.0126	− 0.0053	− 0.0254**	− 0.0098
	(0.0094)	(0.0056)	(0.0113)	(0.0063)
Employees	− 0.0247***	0.0236***	− 0.0008	0.0127
	(0.0064)	(0.0034)	(0.0131)	(0.0096)
Sole proprietorship	0.0317*	− 0.0125	− 0.0271	− 0.0035
	(0.0166)	(0.0110)	(0.0228)	(0.0112)
Domestic	0.0191	0.0412***	− 0.0071	0.1136***
	(0.0253)	(0.0127)	(0.0289)	(0.0154)
Female-led	0.0257*	− 0.0116	0.0071	− 0.0271**
	(0.0139)	(0.0091)	(0.0198)	(0.0113)
Family owned	0.0158	0.0091	− 0.0066	0.0220*
	(0.0129)	(0.0076)	(0.0143)	(0.0114)
Audited	− 0.0344**	0.0076	0.0114	0.0366**
	(0.0142)	(0.0081)	(0.0233)	(0.0161)
Quality certification	− 0.0124	0.0127	0.0204	0.0055
	(0.0144)	(0.0087)	(0.0237)	(0.0153)
Manager experience	0.0070	0.0010	0.0069	0.0074
	(0.0094)	(0.0056)	(0.0124)	(0.0070)
Direct exporter	0.0122	0.0107	0.0423**	0.0424***
	(0.0150)	(0.0086)	(0.0200)	(0.0131)
Trade credit	− 0.0193	0.0308***	− 0.0631***	0.1033***
	(0.0132)	(0.0082)	(0.0227)	(0.0158)
Financial openness	− 0.0290***	0.0178***	− 0.0635***	0.0993***
	(0.0105)	(0.0066)	(0.0239)	(0.0145)
Informal financing	0.0398	0.0437**	0.0861***	0.0341*
	(0.0285)	(0.0174)	(0.0272)	(0.0190)
Large city	0.0311**	− 0.0080	0.0253*	0.0246**
	(0.0158)	(0.0096)	(0.0138)	(0.0110)
Weeks closed	0.0023***	0.0011***		
	(0.0006)	(0.0003)		
Days since pandemic	− 0.0003***	− 0.0002***		
	(0.0001)	(0.0000)		
Stringency index	0.0004	0.0005		
	(0.0007)	(0.0004)		
Internal Financing		− 0.0460***		− 0.0907***
		(0.0068)		(0.0104)
Business obstacles		0.0256***		0.0398***
		(0.0077)		(0.0139)
Country and sector FEs	Yes	Yes	Yes	Yes
Survey round FEs	Yes	Yes	No	No
Wald test of no selectivity bias	[0.0015]		[0.0019]	
Weak-instrument *F* test				
* Green management*	[0.0000]		[0.0000]	
* Credit constraints*	[0.0000]			
Wald test of exogeneity				
* Green management*	[0.0464]	[0.0088]	[0.0198]	[0.0536]
* Credit constraints*	[0.0499]	[0.0489]		
Observations	15,172		8645	

The table reports the average marginal effects on the conditional probability of being denied credit and on the probability of having applied for a loan during and before the COVID-19 pandemic, estimated from pooled endogenous probit models with sample selection. Standard errors, clustered at the firm level, are reported below the estimates. We use *Internal Financing* and *Business Obstacles* as identification variables of the probit model with selection and *Peer green management* and *Peer credit constraints* as additional instruments for the endogenous regressors *Green management* and *Credit constraints*. The *p* values of the Wald tests of no selection bias, of the *F* tests for weak instruments, and of the Wald tests of exogeneity are reported in square brackets

^***^, **, and * denote significance at the 1, 5, and 10% levels, respectively

From the estimated AMEs, reported in columns 1 and 2 of Table 9, we highlight that the quality of green management significantly reduces the probability of applying for credit during the pandemic (by more than 5 percentage points), but does not affect the conditional probability of being denied credit. This evidence is partly in contrast with our research hypothesis HP3 and with the findings of Wellalage et al. (2022a), who show that firms’ environmental performance increases access to bank credit during the COVID-19 crisis. However, it is worth remarking that, differently from Wellalage et al. (2022a) who analyse firms’ recourse to bank financing as the main source used to deal with pandemic-induced liquidity shortages, here we assess the probability of actual credit rationing by banks, addressing both sample selectivity and endogeneity concerns. In doing so, we are able to point out that, during the pandemic, green management quality mainly affects a firm’s credit demand behaviour rather than banks’ lending decisions. This evidence suggests that, since the COVID-19 outbreak, green firms have refrained from applying for bank credit, due to lower financing needs or because they may have been discouraged from applying by unfavourable lending conditions or fear of rejection.

Analysing the impact of prior financing difficulties, we find that those firms that were denied credit or discouraged from applying before the pandemic are about 11.5% more likely to face credit rejection and 10.4% less likely to apply for additional credit during the COVID-19 crisis. Coherently with Pigini et al. (2016) and Aristei & Angori (2022), our results highlight that financing difficulties tend to persist over time, due to observed firm characteristics, unobserved heterogeneity, and true state dependence. We also provide evidence of a significant discouragement effect of prior credit restrictions on firms’ loan demand behaviour during the pandemic, in line with the findings of Cowling et al. (2022a).

With respect to control variables, we find that larger firms have a higher loan demand and are less likely to be denied credit by banks. Consistently with the extant literature (Beck et al., 2006; Ferri et al., 2019), this evidence confirms that smaller businesses are less likely to apply for bank financing, due to their lower financing needs, but face significantly higher difficulties in accessing bank credit, due to their higher informational opacity and lower availability of collateral. Similarly, sole proprietorship firms and those whose financial statements are not certificated by external auditors, being characterized by higher information opacity, are more likely to be rationed when they apply for credit. In line with the results of Wellalage et al. (2022b) and Birindelli et al. (2022), we also point out that female-led businesses are characterized by a significantly higher probability (about 2.6 percentage points) of being credit rationed than their male counterparts. This evidence highlights the heightened financing difficulties experienced by female-led firms since the outbreak of the pandemic and may be indicative of discrimination against women-led businesses by financial institutions during the COVID-19 crisis. We also find that firms with a larger number of products and services contracted with banks are more likely to apply for additional financing and less likely be denied credit. Thus, coherently with previous literature (Angori et al., 2019; Cowling et al., 2016), firms with stable and strong banking relationships are characterized by a higher dependence on bank financing and benefit from enhanced access to credit, especially in crisis periods. Furthermore, we show that firms resorting to trade credit from their supplier and to informal financing before the pandemic are more likely to have applied for bank credit during the pandemic, suggesting complementarity between different financing sources. At the same time, we find that firms mainly relying on internal funds for their working capital needs have a lower demand for credit, while perceived business obstacles increase the likelihood of applying for bank credit, supporting the identifying assumptions underlying our instrumentation strategy. The duration of temporary shutdowns due to COVID-19 restrictions significantly decreases loan demand by 0.1 percentage points and increases rationing probability by 0.2 percentage points for each additional week of temporary closure, respectively. Finally, both credit demand and financing constraints tend to significantly reduce over the course of the pandemic, as firms gradually recover from the COVID-19 shock, while they are not significantly affected by the overall stringency of government containment measures at the country level. As suggested by Wellalage et al. (2022b), this latter evidence can be explained by the counteracting effect of the exceptional government measures to support lending through guaranteed loans and favour firms’ access to finance during the most severe phases of the pandemic.

To assess whether the impact of green management (and other firm-level characteristics) on firms’ financing constraints changed during the pandemic, we re-estimate the endogenous probit model with sample selection to analyse the pre-COVID-19 credit access conditions of the 8645 enterprises analysed in the pandemic period. Results, reported in columns 3 and 4 of Table 9, confirm the endogeneity of the green management score with respect to both credit demand and rationing probabilities. From the estimated AMEs, we find that the quality of firms’ green management significantly enhances access to bank credit in the pre-COVID-19 period, coherently with the findings of Xing et al. (2020), Zhang (2021), and Wellalage & Kumar (2021) and with our predictions (HP3). Specifically, a one standard deviation increase in the green management score raises the probability of applying for credit by 8.7 percentage points, while it decreases the conditional probability of credit rejection by almost12 percentage points. The evidence obtained indicates that, before the pandemic, environmentally sustainable firms had a significantly higher demand for external credit, which was necessary to finance their investment in green technologies (De Haas et al., 2022). Conversely, during the COVID-19 crisis, firms with higher environmental management quality are significantly less likely to apply for credit, possibly suggesting a slowdown in their green investment activities induced by the pandemic. Moreover, in normal market conditions, green management quality strongly improves the likelihood of obtaining bank financing, as firms with sound environmental performance tend to have greater efficiency, profitability, and reputation and are thus considered as less risky and more creditworthy by banks. After the COVID-19 outbreak, firms’ green management practices lose their relevance and do not significantly affect banks’ lending decisions. Taken together, these results suggest that the pandemic shock, by heightening credit constraints and hampering the beneficial role of green management quality on access to credit, may inhibit firms’ investments in green technologies and pollutants abatement and, as pointed out by Guérin & Suntheim (2021), contribute to slow down the transition to a low-carbon economy.

We further point out that, in normal market conditions, domestic, family-owned, and audited firms have a higher propensity to apply for additional credit. We also find that direct exporters and firms located in large cities are characterized by significantly higher loan demand and rationing probabilities. In the period immediately preceding the pandemic, in line with the results of Birindelli et al. (2022), we do not find evidence of significant gender differences in credit rationing probability, while we show that female-led businesses are significantly less likely to apply for credit, due to lower financing needs, but also because they may be discouraged from applying for additional credit. Moreover, firms using trade credit and having a higher financial openness are more likely to apply for credit and have a lower rationing probability before the pandemic’s outbreak, suggesting that access to credit from suppliers and strong banking relationships alleviate financing constraints; conversely, firms recurring to informal have limited access to finance, as they may be perceived as riskier by banks.

## Concluding remarks

In this paper, we investigate the consequences of the COVID-19 crisis on firms’ economic performance, financial vulnerability, and access to credit. We carry out our empirical analysis using longitudinal firm-level data from the World Bank’s “Enterprise Surveys follow-up on COVID-19” for 20 European countries and assess whether green management quality and pre-pandemic credit access difficulties affect firms’ resilience to the impact of the pandemic shock.

Consistently with our research hypotheses, we provide robust empirical evidence indicating that green firms are less vulnerable to the pandemic shock. Specifically, firms adopting high-quality green management practices are found to experiment a lower probability of a decline in sales and are exposed to lower liquidity issues. We further demonstrate that previous financing restrictions strongly exacerbate the negative impact of the COVID-19 shock. Credit-constrained firms are not only more likely to experience cash flow shortages and delays in payments and financial obligations, but they also face higher difficulties in accessing bank credit during the pandemic. Finally, we show that the quality of green management does not affect the probability of access to credit during the pandemic and firms with sound environmental management have a lower demand for credit, suggesting a foreseeable slowdown in their green investment activities.

Our empirical findings offer some relevant implications both for firms and policymakers. In particular, public policies and programmes aimed at encouraging firms’ environmentally sustainable behaviours may strongly contribute to improve the resilience of the economic system to unexpected shocks. Furthermore, policy initiatives supporting firms’ access to credit may foster their capacity to absorb shocks and, at the same time, favour the transition to a sustainable economy in the long run. From the firms’ perspective, the awareness of the competitive advantages offered by eco-sustainable practices, in terms of better performance and lower financial vulnerability, may encourage enterprises to improve environmental management quality and increase investment in green technologies, strengthening their ability to withstand adverse shocks and setting out a path to achieve strong and environmentally sustainable economic growth.

## Electronic supplementary material

Below is the link to the electronic supplementary material.

Supplementary file1 (DOCX 1817 KB)

## Data Availability

The raw firm-level data used in this study are available from the World Bank's Enterprise Surveys data portal at www.enterprisesurveys.org.

## Appendix

Table

**Table 10:** Firms’ distribution by country and sector

	Sector			
Country	Manufacturing	Retail	Services	Total
Albania	130	72	141	343
Bulgaria	353	106	174	633
Croatia	108	76	125	309
Cyprus	75	55	85	215
Czech Republic	268	55	137	460
Estonia	125	72	136	333
Georgia	197	110	241	548
Greece	287	119	149	555
Hungary	440	126	168	734
Italy	345	76	108	529
Latvia	122	93	125	340
Lithuania	110	92	95	297
Malta	77	51	97	225
Moldova	125	88	110	323
North Macedonia	122	99	103	324
Poland	822	91	210	1123
Portugal	648	103	133	884
Romania	403	97	145	645
Slovak Republic	162	89	104	355
Slovenia	143	62	139	344
Total	5062	1732	2725	9519

**Table 11:** Variable definitions and descriptive statistics

Variable	Definition	Mean
(a) Dependent variables		
Sales decreased	Equals 1 if the firm has experienced a decrease in sales compared with the same month of the previous year (i.e. 2019 for those interviewed in 2020; 2020 for those interviewed in 2021); 0 otherwise	0.5343
Sales change	Percentage change in firm sales compared with the same month of the previous year (i.e. 2019 for those interviewed in 2020; 2020 for those interviewed in 2021)	− 16.40
Expected recovery	Expected time needed for the firm’s sales to get back to normal. Ordinal variable with 4 categories: 0 “current sales are as normal”, 1 “1 to 6 months”, 2 “7 to 12 months”; 3 “more than 12 months/never”	0.8862
Liquidity decreased	Equals 1 if the firm has experienced a decrease in liquidity or cash flow since the outbreak of COVID-19 or since the previous round of the WBES COVID-19 Follow-up Survey (for firms interviewed in more than one round); 0 otherwise	0.4748
Delayed payments	Equals 1 if the firm has delayed payments to its suppliers, its landlords, or tax authorities for more than 1 week (excluding payments postponed following current regulation) since the outbreak of COVID-19 or since the previous round of the WBES COVID-19 Follow-up Survey (for firms interviewed in more than one round); 0 otherwise	0.2812
Financial obligations overdue	Equals 1 if the firm has been overdue on its obligations to any financial institution since the outbreak of COVID-19 or since the previous round of the WBES COVID-19 Follow-up Survey (for firms interviewed in more than one round); 0 otherwise	0.1014
Applied credit	Equals 1 if the firm has applied for any line of credit or loan since the outbreak of COVID-19 or since round 2 of the WBES COVID-19 Follow-up Survey (for firms re-interviewed in round 3); 0 otherwise	0.1896
Credit denied	Equals 1 if the firm’s most recent application for a line of credit or loan during the COVID pandemic was rejected; 0 if it was approved	0.1336
(b) Explanatory variables		
Green management	*z*-score based on the firm’s green management practices in four areas: strategic objectives related to the environment and climate change, manager with explicit mandate to deal with green issues, setting environmental targets, monitoring environmental targets	0.0000
Credit constraints	Equals 1 if, in the last WBES completed before to the COVID-19 outbreak, the firm declared that it applied for a line of credit or a loan and was denied credit or that it needed a loan, but it did not apply due to unfavourable credit conditions and/or fear of rejection; 0 otherwise	0.1162
Age	Years since the firm’s establishment (in logs)	2.7504
Employees	Number of full-time employees (in logs)	2.5852
Sole proprietorship	Equals 1 if the firm’s legal status is sole proprietorship; 0 otherwise	0.3671
Domestic	Equals 1 if the firm’s first shareholder is domestic; 0 otherwise	0.9591
Female-led	Equals 1 if the firm’s top manager is a woman; 0 otherwise	0.2306
Family owned	Equals 1 if at least 50% of the same family; 0 otherwise	0.5065
Audited	Equals 1 if the firm has its financial statement certified by external auditors, 0 otherwise	0.1417
Quality certification	Equals 1 if the firm has an internationally recognized quality certification, 0 otherwise	0.1937
Manager experience	Top manager’s years of experience in the sector (in logs)	2.8350
Direct exporter	Equals 1 if the firm directly exports its production abroad; 0 otherwise	0.1033
Trade credit	Equals 1 if the firm purchases on credit material inputs or services; 0 otherwise	0.5013
Financial openness	Number of products and services contracted with banks	1.3985
Informal financing	Equals 1 if the firm uses informal finance (i.e. moneylenders, friends, relatives) to purchase fixed assets or for its working capital financing; 0 otherwise	0.0323
Internal financing	Equals 1 if the firm uses internal funds or retained earnings as the main source of its working capital financing; 0 otherwise	0.6723
Large city	Equals 1 if the firm is located in a capital or a large city (more than 1 million inhabitants); 0 otherwise	0.1690
Weeks closed	Numbers of weeks the firm was temporarily closed due to the COVID-19 outbreak	0.7597
Days since pandemic	Days between the day of interview and 11 March 2020, day in which the World Health Organization (WHO) declared COVID-19 as a pandemic	279.00
Stringency index	Country-level composite index based on nine daily indicators of government response initiatives to address the spreading of the coronavirus (school closures, workplace closures, cancellation of public events, restrictions on gatherings, public transport closures, stay-at-home requirements, restrictions on internal movements, international travel bans, and public information campaigns), rescaled to range between 0 and 100 (with higher values indicating stricter restrictions). Source: Oxford COVID-19 Government Response Tracker, Blavatnik School of Government, University of Oxford	55.855
General management	*z*-score based on the firm’s general management practices in four areas: operations, monitoring, targeting, and incentives. This variable is available only for firms with at least 20 employees	0.0000
Business obstacles	Equals 1 if the firm perceives business licencing, trade regulations, corruption, tax rates, and administration as significant obstacles to its operations; 0 otherwise	0.8366
Peer green management	Average values of the *Green management z*-score computed on the other enterprises operating in the same region of the firm	-0.0173
Peer credit constraints	Average value of the *Credit* constraints binary indicatory computed on the other enterprises operating in the same region of the firm, excluding those operating in the same sector	0.1122

Average values are computed using sample weights, rescaled by the inverse of the number of observations in each country

## Footnotes

1 Supplementary Appendix Table S1 reports details on the survey implementation periods in each country.

2 Supplementary Appendix Table S2 reports complete details on the “Green Economy Module” questions of the WBES.

3 Since OxCGRT data are not available for North Macedonia, we replace the *Stringency index* for this country with the average value of the index in neighbouring countries.

4 It is worth remarking that ignoring the endogeneity of *Green management* and *Credit constraints* leads to severely downwardly biased estimates of the effects of the two regressors on the probability of a decrease in sales. Specifically, when both the regressors are assumed to be exogenous in Eq. (1), the estimated marginal effect of a unit change in the green management score is equal to − 0.0106, and the discrete effect of the binary indicator of credit constraints is equal to 0.0516. Both these effects, despite remaining statistically significant, are about six and four times lower than those obtained from the endogenous probit model, respectively. We obtain downwardly biased estimates also from the other models considered in our empirical analysis when the endogeneity of *Green management* and *Credit constraints* is not properly addressed. Marginal effects estimated from these exogenous models are reported in Supplementary Appendix Table S5.

5 Supplementary Appendix Fig. S2 shows how the predicted probability of a decrease in sales varies as the number of weeks of temporary closures ranges from 0 and 48 (i.e. the 1st and 99th percentiles of its distribution observed in the whole estimation sample), as the days since the onset of the COVID-19 pandemic ranges from 0 to 660, and as the *Stringency index* ranges from 0 to 100. Similar predictive margins plots are defined for the other empirical analyses and presented in Supplementary Appendix Figs. S3, S4, and S5.

6 We have also tested whether green management quality impacts directly on firms’ economic and financial performance during the COVID19 crisis and indirectly through access to finance. To this aim, as in Mancusi et al. (2018), we have extended the reduced form probit equation for ${Credit constraints}_{i0}$ (3) to include ${Green management}_{i0}$ as an additional regressor. This allows us to separately identify both the direct effect of green management on performance and the indirect effect passing through financing constraints. Results (available from the authors) show that the indirect effect is never statistically significant, so that the effect of green management on performance is direct and not mediated by pre-pandemic financing difficulties.

7 Supplementary Appendix Table S6 reports complete details on the questions in the “Management practices” section of the WBES. Supplementary Appendix Fig. S6 shows the distribution of the *General Management* score in each country.

8 In particular, the estimated correlation coefficient is equal to − 0.8672. As discussed in Brown et al. (2011) and Aristei and Gallo (2022), the negative value of cross-equation correlation suggests that firms which are more likely to have an application rejected are also less likely to apply as they anticipate rejection and prefer to remain out of the credit market.
