# Supplementary material for: Green management, access to credit, and firms’ vulnerability to the COVID-19 crisis
Source: Small Bus Econ (Dordr). 2023 Apr 19:1–33. Online ahead of print. doi: 10.1007/s11187-023-00759-1 (PMC10113997; doi:10.1007/s11187-023-00759-1)
Supplement: Supplementary file 1 — Supplementary file1 (DOCX 1817 KB) [file 11187_2023_759_MOESM1_ESM.docx]

Green management, access to credit

and firms’ vulnerability to the COVID-19 crisis

**Supplementary Appendix**

Tables

Table S1 – WBES and WBES-COVID implementation periods by country

| Country | WBES | WBES-COVID Round 1 | WBES-COVID Round 2 | WBES-COVID Round 3 |
| --- | --- | --- | --- | --- |
|  |  |  |  |  |
| Albania | January/May 2019 | June 2020 | – | – |
| Bulgaria | January/March 2020 | July/September 2020 | November/December 2020 | April/May 2021 |
| Croatia | November 2018/ November 2019 | September 2020 | December 2020/January 2021 | May/June 2021 |
| Cyprus | December 2018/June 2019 | June 2020 | November/December 2020 | April 2021 |
| Czech Republic | April/November 2019 | June 2020 | October/November 2020 | May/June 2021 |
| Estonia | November 2018/January 2020 | October 2020 | February 2021 | July/August 2021 |
| Georgia | March 2019/January 2020 | June 2020 | October/November 2020 | September/October 2021 |
| Greece | September 2018/April 2019 | June 2020 | November 2020 | April/May 2021 |
| Hungary | December 2018/March 2020 | September 2020 | January/February 2021 | May/June 2021 |
| Italy | November 2018/October 2019 | June 2020 | November/December 2020 | April/May 2021 |
| Latvia | November 2018/December 2019 | September 2020 | February 2021 | July/August 2021 |
| Lithuania | December 2018/January 2020 | October 2020 | February 2021 | July/August 2021 |
| Malta | December 2018/September 2019 | September 2020 | January 2021 | May 2021 |
| Moldova | April/November 2019 | June 2020 | October/November 2020 | May/June 2021 |
| North Macedonia | December 2018/October 2019 | October/November 2020 | May/June 2021 | December 2021/January 2022 |
| Poland | December 2018/December 2019 | July/August 2020 | November/December 2020 | May/June 2021 |
| Portugal | November 2018/January 2020 | September/October 2020 | January/February 2021 | May/June 2021 |
| Romania | December 2018/June2020 | August/September 2020 | November/December 2020 | April/June 2021 |
| Slovak Republic | December 2018/March 2020 | September/October 2020 | January/February 2021 | May/June 2021 |
| Slovenia | December 2018/November 2019 | July/August 2020 | November/December 2020 | May/June 2021 |
|  |  |  |  |  |

Table S2 – Questions in the “Green Management Module” of the WBES

| Green managerial area | Question |
| --- | --- |
|  |  |
| a) Strategic objectives | 1) BMGA1: *In the last fiscal year, did this firm have strategic objectives that mention environmental or climate change issues?* |
|  |  |
| b) Managers' responsibility | 1) BMGA2: *In the last fiscal year, did this establishment have a manager responsible for environmental and climate change issues?* |
|  | 2) BMGA3: *In the last fiscal year, whom did the manager responsible for environmental and climate change issues directly report to?* |
|  | 3) BMGC22: *Is the manager responsible for environmental and climate change issues evaluated against how well the establishment performs on energy consumption, CO2 emissions or other pollution or environmental targets?* |
|  |  |
| c) Monitoring | 1) BMGC1: *Over the last three years, did this establishment monitor its energy consumption?* |
|  | 2) BMGC3: *Over the last three years, did this establishment complete an external audit of its energy consumption?* |
|  | 3) BMGC4: *Over the last three years, did this establishment monitor its water usage?* (Manufacturing firms only) |
|  | 4) BMGC6: *Over the last three years, did this establishment complete an external audit of its water usage*? (Manufacturing firms only) |
|  | 5) BMGC8: *Over the last three years, did this establishment monitor its CO2 emissions?* |
|  | 6) BMGC10: *Over the last three years, did this establishment complete an external audit of its CO2 emissions?* |
|  | 7) BMGC11: *Over the last three years, did this establishment monitor CO2 emissions along its supply chain?* |
|  | 8) BMGC13: *Over the last three years, did this establishment monitor its emissions of pollutants other than CO2?* (Manufacturing firms only) |
|  | 9) BMGC15: *Over the last three years, did this establishment complete an external audit of its pollutants other than CO2?* (Manufacturing firms only) |
|  |  |
| d) Targeting | 1) BMGC16: *Over the last three years, did this establishment have targets for energy consumption?* |
|  | 2) BMGC18: *Over the last three years, did this establishment have targets for CO2 emissions?* |
|  | 3) BMGC20: *Over the last three years, did this establishment have targets for pollution emissions other than CO2?* (Manufacturing firms only) |
|  |  |

Table S3 – Correlation matrix of the explanatory variables

|  |  | (1) | (2) | (3) | (4) | (5) | (6) | (7) | (8) | (9) | (10) | (11) |
| --- | --- | --- | --- | --- | --- | --- | --- | --- | --- | --- | --- | --- |
|  |  |  |  |  |  |  |  |  |  |  |  |  |
| (1) | Green management | 1.000 |  |  |  |  |  |  |  |  |  |  |
| (2) | Credit constraints | 0.083 | 1.000 |  |  |  |  |  |  |  |  |  |
| (3) | Age | 0.112 | -0.025 | 1.000 |  |  |  |  |  |  |  |  |
| (4) | Employees | 0.253 | 0.024 | 0.199 | 1.000 |  |  |  |  |  |  |  |
| (5) | Sole Proprietorship | -0.150 | -0.096 | -0.124 | -0.271 | 1.000 |  |  |  |  |  |  |
| (6) | Domestic | -0.075 | 0.036 | 0.062 | -0.151 | 0.095 | 1.000 |  |  |  |  |  |
| (7) | Female-led | -0.034 | 0.009 | -0.112 | -0.116 | 0.098 | -0.023 | 1.000 |  |  |  |  |
| (8) | Family owned | 0.035 | -0.009 | 0.068 | -0.030 | 0.013 | 0.074 | 0.051 | 1.000 |  |  |  |
| (9) | Audited | 0.220 | 0.009 | 0.071 | 0.288 | -0.195 | -0.139 | -0.079 | 0.048 | 1.000 |  |  |
| (10) | Quality certification | 0.132 | 0.102 | 0.148 | 0.272 | -0.216 | -0.055 | -0.082 | 0.109 | 0.194 | 1.000 |  |
| (11) | Manager experience | 0.004 | -0.102 | 0.485 | 0.083 | -0.102 | 0.081 | -0.174 | 0.126 | 0.062 | 0.140 | 1.000 |
| (12) | Direct exporter | 0.169 | -0.043 | 0.074 | 0.272 | -0.152 | -0.154 | -0.065 | 0.097 | 0.202 | 0.172 | 0.075 |
| (13) | Trade credit | 0.049 | 0.095 | -0.004 | 0.120 | -0.053 | 0.059 | -0.049 | 0.084 | 0.081 | 0.121 | 0.068 |
| (14) | Financial openness | 0.111 | -0.005 | 0.079 | 0.169 | -0.080 | 0.004 | -0.101 | 0.048 | 0.103 | 0.177 | 0.100 |
| (15) | Informal financing | 0.071 | 0.086 | -0.020 | 0.012 | -0.016 | 0.005 | -0.029 | 0.046 | 0.017 | -0.012 | -0.067 |
| (16) | Large city | 0.011 | -0.021 | 0.046 | 0.012 | -0.138 | -0.030 | -0.088 | 0.022 | 0.077 | 0.039 | -0.018 |
| (17) | Weeks closed | -0.006 | -0.001 | -0.088 | -0.041 | 0.015 | 0.025 | 0.088 | 0.027 | -0.027 | -0.030 | -0.081 |
| (18) | Days since pandemic | 0.001 | -0.024 | -0.009 | -0.001 | 0.033 | -0.007 | 0.036 | 0.022 | 0.010 | -0.067 | -0.018 |
| (19) | Stringency index | 0.002 | 0.063 | 0.028 | 0.014 | -0.029 | 0.045 | -0.021 | 0.040 | -0.007 | 0.150 | 0.033 |
| (20) | Internal Financing | 0.007 | -0.148 | 0.061 | -0.009 | 0.051 | 0.002 | -0.057 | 0.117 | 0.040 | 0.026 | 0.080 |
| (21) | Business Obstacles | 0.101 | 0.059 | -0.027 | -0.038 | 0.009 | 0.005 | 0.059 | 0.019 | -0.030 | 0.004 | -0.084 |
|  |  |  |  |  |  |  |  |  |  |  |  |  |
|  |  | (12) | (13) | (14) | (15) | (16) | (17) | (18) | (19) | (20) | (21) |  |
|  |  |  |  |  |  |  |  |  |  |  |  |  |
| (12) | Direct exporter | 1.000 |  |  |  |  |  |  |  |  |  |  |
| (13) | Trade credit | 0.042 | 1.000 |  |  |  |  |  |  |  |  |  |
| (14) | Financial openness | 0.094 | 0.252 | 1.000 |  |  |  |  |  |  |  |  |
| (15) | Informal financing | 0.053 | 0.038 | -0.005 | 1.000 |  |  |  |  |  |  |  |
| (16) | Large city | 0.014 | 0.025 | -0.006 | 0.052 | 1.000 |  |  |  |  |  |  |
| (17) | Weeks closed | -0.040 | -0.007 | 0.003 | -0.013 | 0.014 | 1.000 |  |  |  |  |  |
| (18) | Days since pandemic | 0.013 | -0.045 | -0.043 | -0.002 | 0.014 | 0.096 | 1.000 |  |  |  |  |
| (19) | Stringency index | -0.030 | 0.055 | 0.071 | -0.015 | -0.041 | 0.046 | 0.273 | 1.000 |  |  |  |
| (20) | Internal Financing | 0.053 | -0.169 | -0.047 | -0.049 | 0.061 | -0.006 | 0.038 | -0.016 | 1.000 |  |  |
| (21) | Business Obstacles | -0.003 | 0.060 | 0.005 | -0.012 | 0.032 | 0.009 | -0.018 | 0.055 | -0.011 | 1.000 |  |
|  |  |  |  |  |  |  |  |  |  |  |  |  |

**Notes**: the Table reports pairwise correlation coefficients computed on the estimation sample, computed using sample weights, rescaled by the inverse of the number of observations in each country.

Table S4 – Firms’ green management and credit constraints: estimated AMEs

| Dependent variable: | Green management | Credit constraints |
| --- | --- | --- |
|  | (1) | (2) |
|  |  |  |
| Age (in logs) | 0.0695*** | -0.0040 |
|  | (0.0153) | (0.0051) |
| Employees (in logs) | 0.1737*** | -0.0146*** |
|  | (0.0101) | (0.0032) |
| Sole Proprietorship | -0.0601** | -0.0097 |
|  | (0.0262) | (0.0099) |
| Domestic | -0.1776*** | 0.0152 |
|  | (0.0395) | (0.0122) |
| Female-led | -0.0428* | 0.0067 |
|  | (0.0224) | (0.0080) |
| Family owned | 0.0062 | -0.0001 |
|  | (0.0215) | (0.0068) |
| Audited | 0.2256*** | -0.0148* |
|  | (0.0233) | (0.0076) |
| Quality certification | 0.2723*** | 0.0011 |
|  | (0.0254) | (0.0079) |
| Manager experience | -0.0247 | -0.0027 |
|  | (0.0161) | (0.0052) |
| Direct exporter | 0.1624*** | 0.0011 |
|  | (0.0259) | (0.0082) |
| Trade credit | 0.0427* | 0.0043 |
|  | (0.0220) | (0.0074) |
| Financial openness | 0.0734*** | -0.0096 |
|  | (0.0188) | (0.0059) |
| Informal financing | 0.1026* | 0.0742*** |
|  | (0.0537) | (0.0145) |
| Internal Financing | 0.0336 | -0.0148** |
|  | (0.0223) | (0.0071) |
| Large city | -0.0236 | -0.0139 |
|  | (0.0255) | (0.0090) |
| Business Obstacles | 0.1074*** | 0.0579*** |
|  | (0.0228) | (0.0087) |
| Peer green management | 0.6620*** |  |
|  | (0.0416) |  |
| Peer credit constraints |  | 0.3632*** |
|  |  | (0.0416) |
|  |  |  |
|  |  |  |
| Country and Sector FEs | Yes | Yes |
| Survey round FEs | Yes | Yes |
|  |  |  |
|  |  |  |
| Observations | 23,147 | 23,147 |
|  |  |  |

**Notes:** the Table reports the average marginal effects on the expected value of the green management score and on the probability of facing credit constraints, estimated from the reduced form equations (2) and (3) in the endogenous probit model with a recursive structure for the probability of a decrease in sales. Standard errors, clustered at the firm level, are reported below the estimates.

***, ** and * denote significance at the 1, 5 and 10% levels, respectively.

Table S5 – Exogenous models: Estimated AMEs

|  |  | | | | |
| --- | --- | --- | --- | --- | --- |
|  | *Panel A: Firms’ sales during the COVID-19 pandemic* | | | | |
| Dependent variable: | Sales  decreased | Sales  change |  | |  |
|  |  |  |  | |  |
| Green management | -0.0106** | 0.9782** |  | |  |
|  | (0.0051) | (0.4504) |  | |  |
| Credit constraints | 0.0516*** | -4.2064*** |  | |  |
|  | (0.0117) | (0.7419) |  | |  |
|  |  |  |  | |  |
| Observations | 23,147 | 22,945 |  | |  |
|  |  |  |  | |  |
|  |  |  |  | |  |
|  | *Panel B: Severity of Covid-19 impact on firms* | | | | |
| Dependent variable: | Expected recovery | | | | |
| Ordered outcome: | Current sales as normal | 1 to 6  months | | 7 to 12  months | More than 12 months/never |
|  |  |  | |  |  |
| Green management | 0.0111** | -0.0028** | | -0.0044** | -0.0040** |
|  | (0.0044) | (0.0013) | | (0.0026) | (0.0022) |
| Credit constraints | -0.0548*** | 0.0137*** | | 0.0215*** | 0.0195*** |
|  | (0.0119) | (0.0030) | | (0.0047) | (0.0042) |
|  |  |  | |  |  |
| Observations | 20,468 | | | | |
|  |  |  | |  |  |
|  |  |  | |  |  |
|  | *Panel C: Firms’ financial fragility since the COVID-19 outbreak* | | | | |
| Dependent variable: | Liquidity  decreased | Delayed  payments | | Financial obligations overdue |  |
|  |  |  | |  |  |
| Green management | -0.0126** | 0.0002 | | -0.0014 |  |
|  | (0.0051) | (0.0039) | | (0.0021) |  |
| Credit constraints | 0.0568*** | 0.0545*** | | 0.0263*** |  |
|  | (0.0193) | (0.0121) | | (0.0055) |  |
|  |  |  | |  |  |
| Observations | 23,147 | 21,431 | | 22,320 |  |
|  |  |  | |  |  |

**Notes:** the Table reports the average marginal effects estimated from the regression models presented in Sections 4.1 and 4.2, treating the *Green management* z-score and the binary indicator *Credit constraints* as exogenous regressors. Standard errors, clustered at the firm level, are reported below the estimates. All the regressions include the controls used in Tables 3, 4, and 5. Complete estimation results are available upon request.

***, ** and * denote significance at the 1, 5 and 10% levels, respectively.

Table S6 – Questions in the “Management practices” section of the WBES

| Managerial area | Question |
| --- | --- |
|  |  |
| a) Operations | 1) BMR1: *Over the last complete fiscal year, what best describes what happened at this establishment when a problem in the production process arose?* |
|  |  |
| b) Monitoring | 1) BMR2: *Over the last complete fiscal year, did this establishment monitor any performance indicators?* |
|  | 2) BMR3: *Over the last complete fiscal year, how many performance indicators were monitored at this establishment?* |
|  |  |
| c) Targets | 1) BMR4: *Over the last complete fiscal year, did this establishment have production targets? Examples of production targets are: production volume, quality, efficiency, waste, or on-time delivery.* |
|  | 2) BMR5: *Over the last complete fiscal year, what best describes the time frame of production targets at this establishment?* |
|  | 3) BMR6: *Over the last complete fiscal year, how easy or difficult was it for this establishment to achieve its production targets overall?* |
|  | 4) BMR7: *Over the last complete fiscal year, who was aware of the production targets at this establishment?* |
|  |  |
| d) Incentives | 1) BMR8: *Over the last complete fiscal year, did this establishment have performance bonuses for managers?* |
|  | 2) BMR9: *Over the last complete fiscal year, what were managers' performance bonuses mostly based on?* |
|  | 3) BMR10: *Over the last complete fiscal year, what was the primary way non-managers were promoted at this establishment?* |
|  | 4) BMR11: *Over the last complete fiscal year, when was an under- performing non-manager reassigned or dismissed?* |
|  |  |

**Notes:** questions of the “Management practices” section of the WBES are asked only to firms with at least 20 employees.

Figures


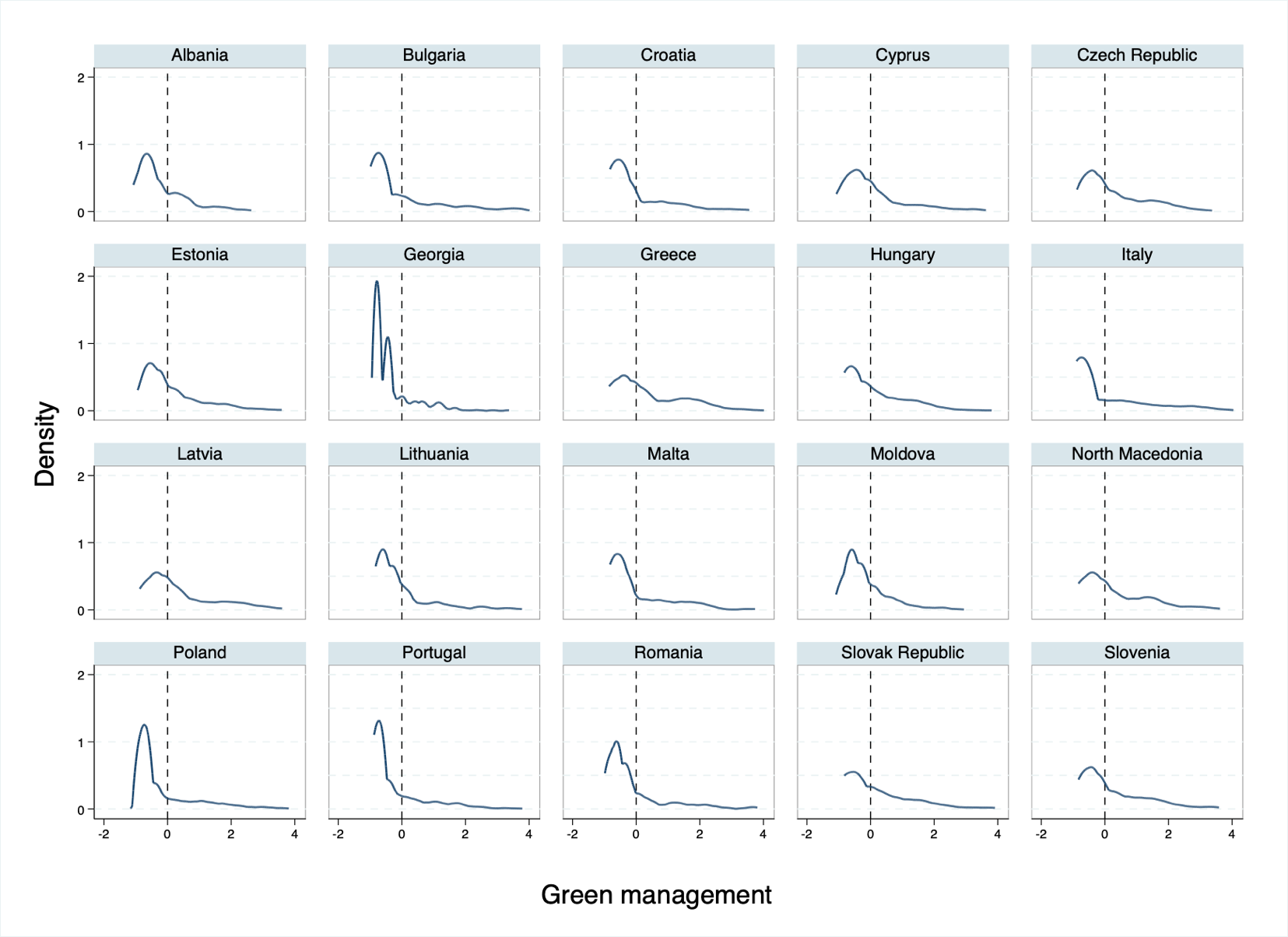


Figure S1 – Distribution of the Green Management score by country

| 1) Probability of a decrease in sales | | |
| --- | --- | --- |
| a) Weeks of temporary closure | b) Days since WHO pandemic declaration | c) Stringency index |
| 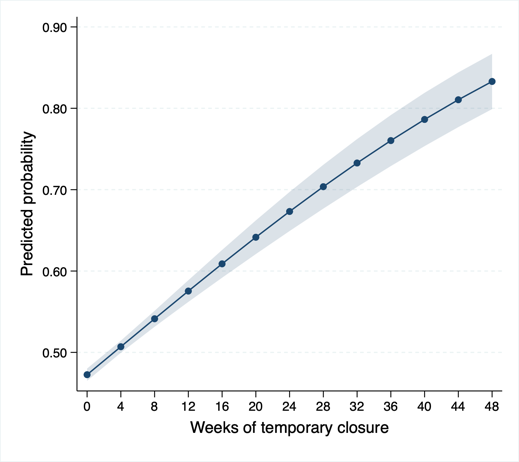 | 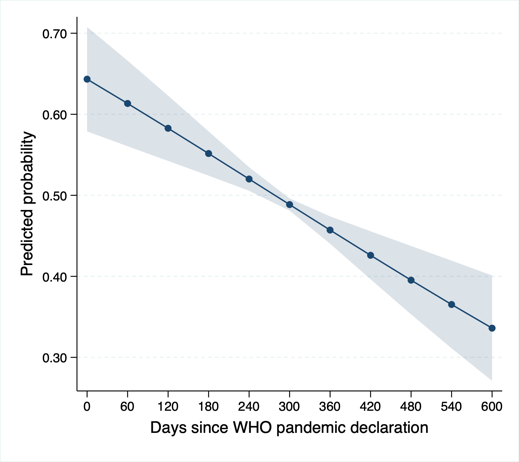 | 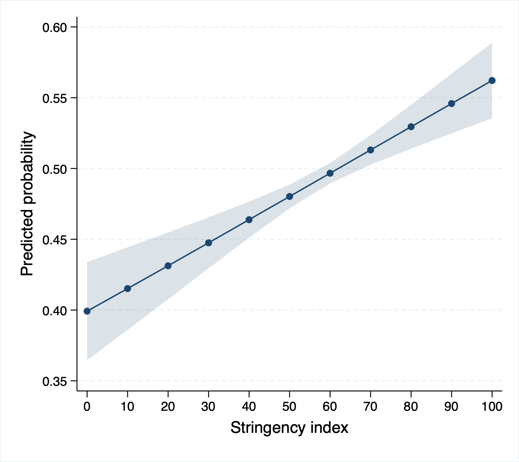 |
|  | | |
| 2) Percentage change in sales | | |
| a) Weeks of temporary closure | b) Days since WHO pandemic declaration | c) Stringency index |
| 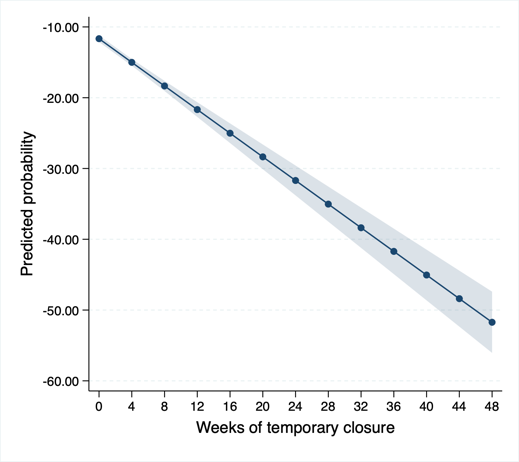 | 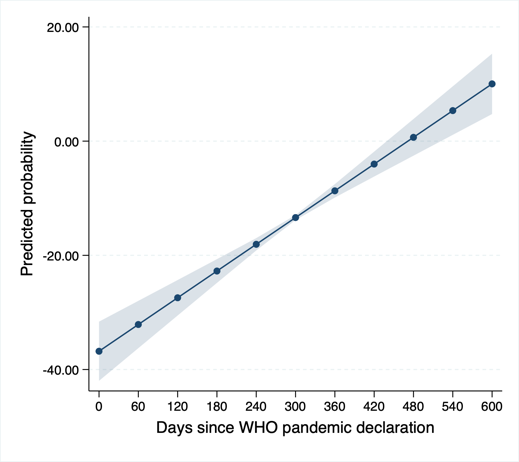 | 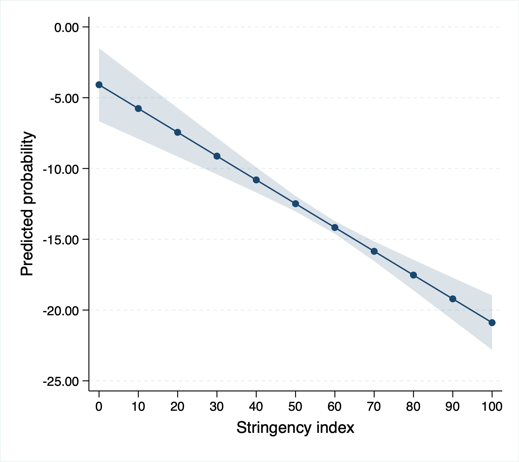 |

Figure S2 – Predicted probability of sales decrease and predicted percentage change in sales

| 1) Current sales are as normal | | |
| --- | --- | --- |
| a) Weeks of temporary closure | b) Days since WHO pandemic declaration | c) Stringency index |
| 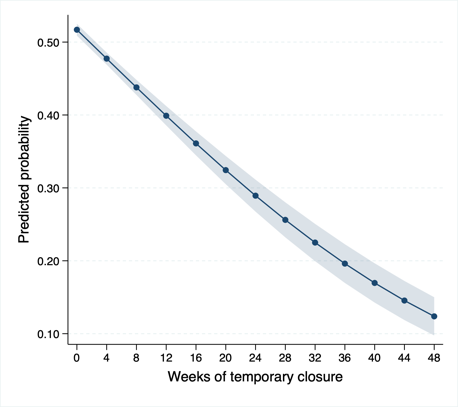 | 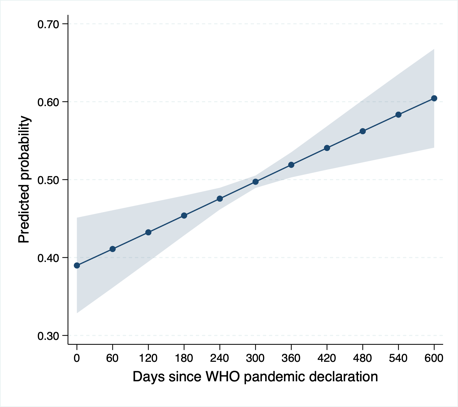 | 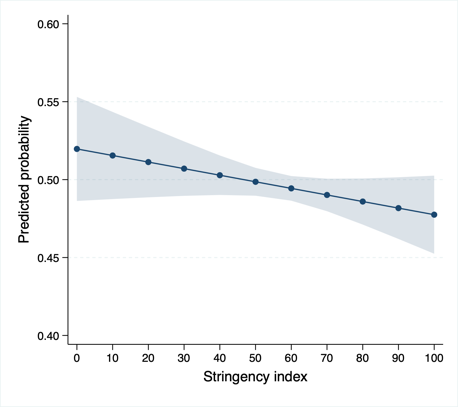 |
|  | | |
| 2) Recover in 1 to 6 months | | |
| a) Weeks of temporary closure | b) Days since WHO pandemic declaration | c) Stringency index |
| 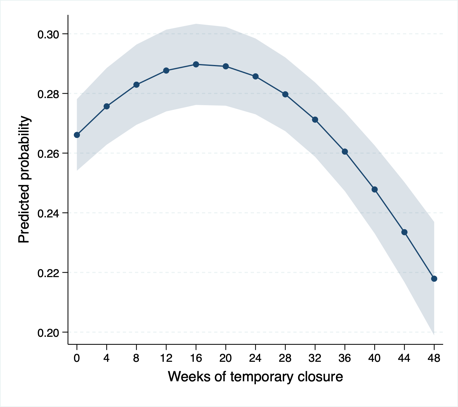 | 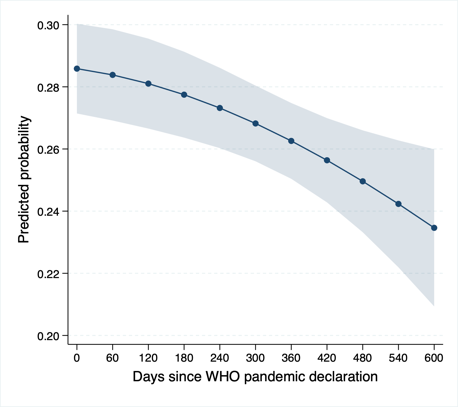 | 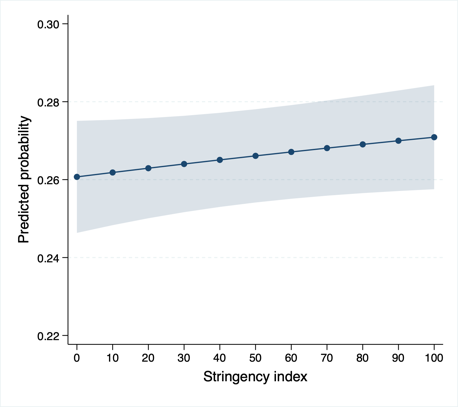 |
|  | | |
| 3) Recover in 7 to 12 months | | |
| a) Weeks of temporary closure | b) Days since WHO pandemic declaration | c) Stringency index |
| 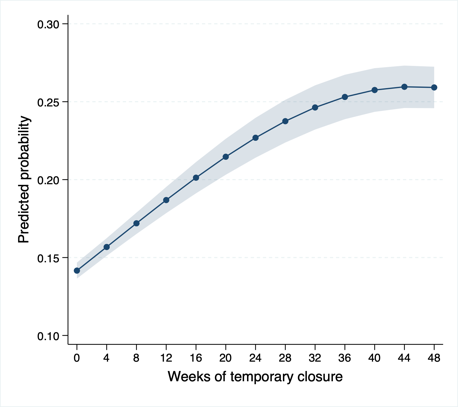 | 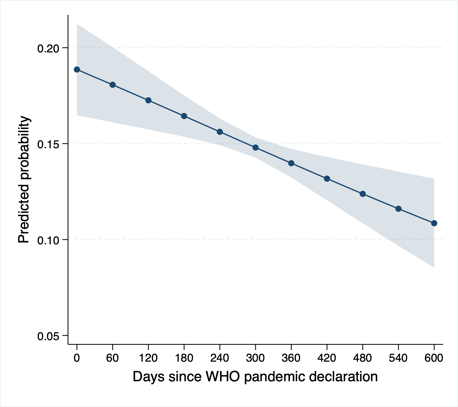 | 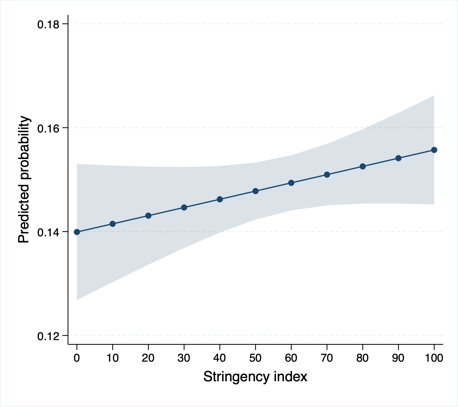 |
|  | | |
| 4) Recover in more than 12 months/never | | |
| a) Weeks of temporary closure | b) Days since WHO pandemic declaration | c) Stringency index |
| 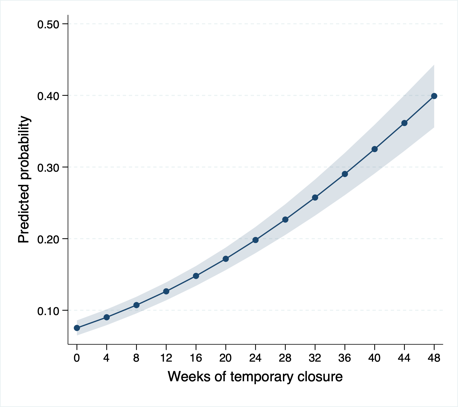 | 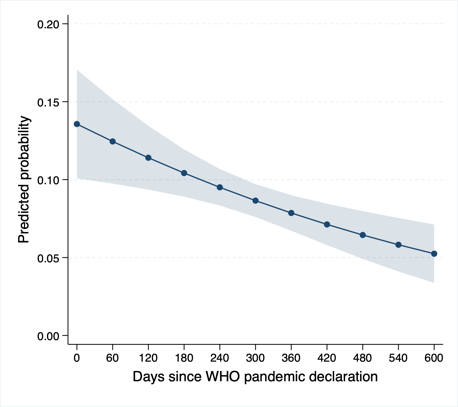 | 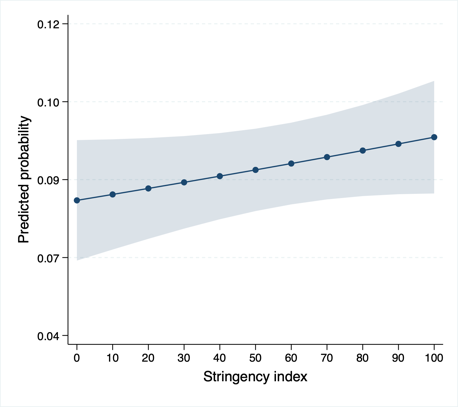 |

Figure S3 – Predicted probabilities of expected time to recover from the COVID-19 shock

| 1) Decrease in liquidity | | |
| --- | --- | --- |
| a) Weeks of temporary closure | b) Days since WHO pandemic declaration | c) Stringency index |
| 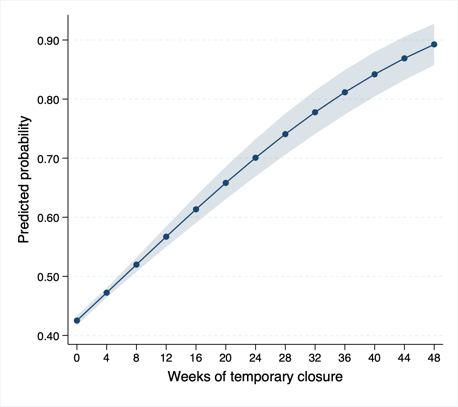 | 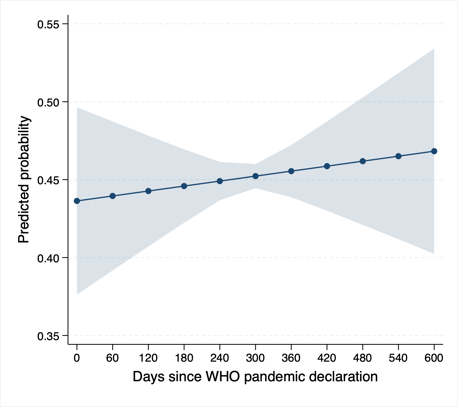 | 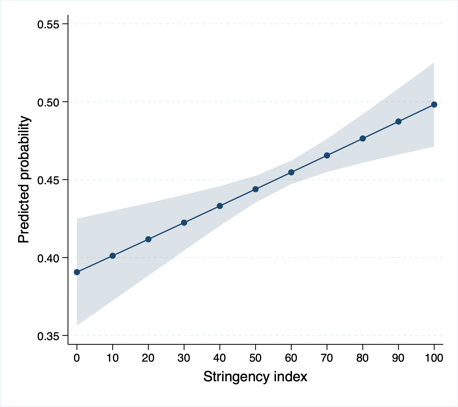 |
|  | | |
| 2) Delayed payments | | |
| a) Weeks of temporary closure | b) Days since WHO pandemic declaration | c) Stringency index |
| 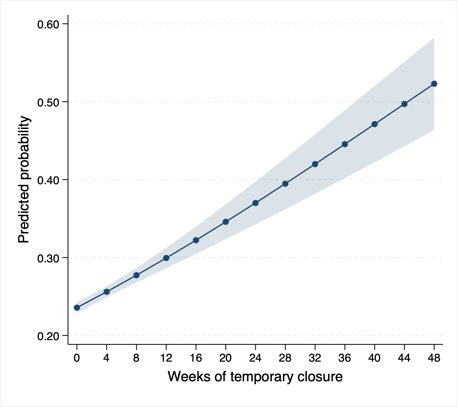 | 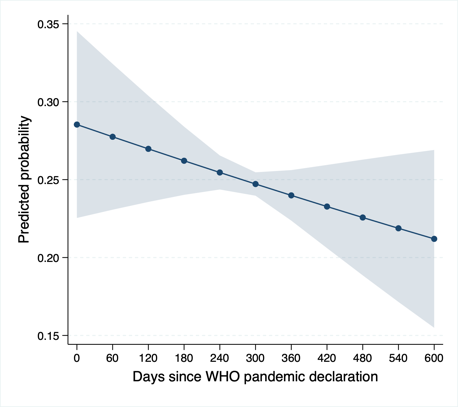 | 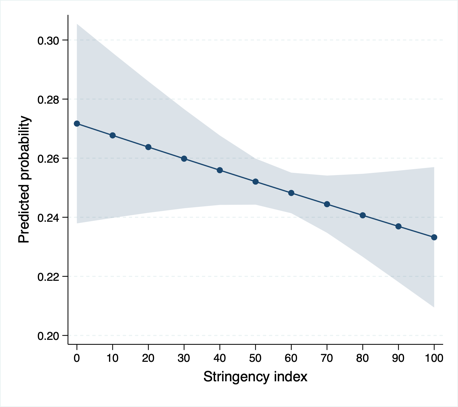 |
|  | | |
| 3) Financial obligations overdue | | |
| a) Weeks of temporary closure | b) Days since WHO pandemic declaration | c) Stringency index |
| 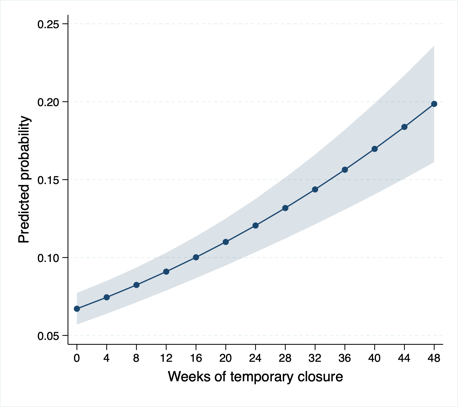 | 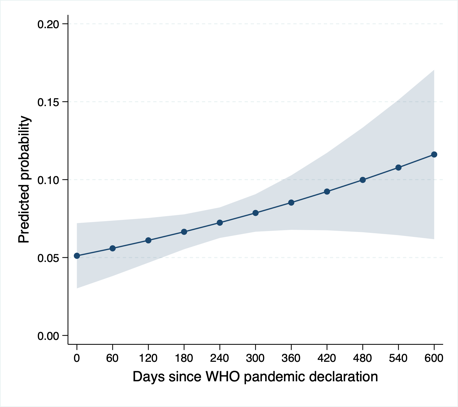 | 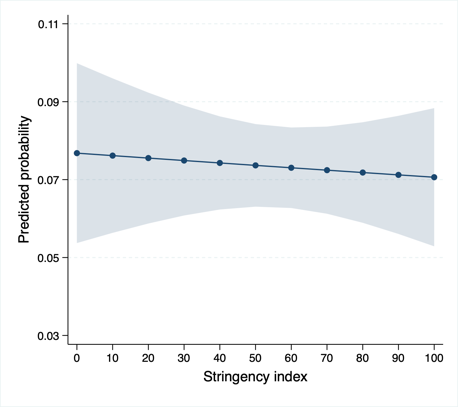 |

Figure S4 – Predicted probabilities of experiencing different pandemic-induced financial issues

| 1) Credit rationing | | |
| --- | --- | --- |
| a) Weeks of temporary closure | b) Days since WHO pandemic declaration | c) Stringency index |
| 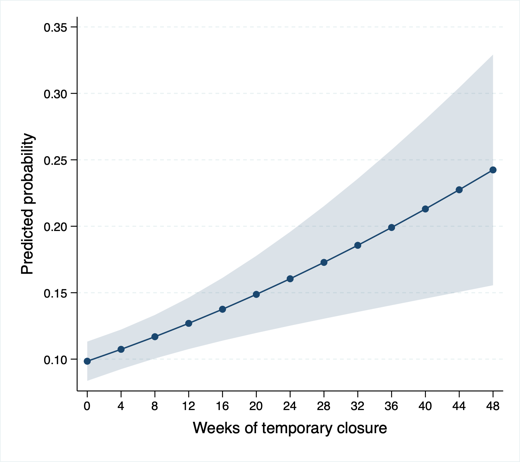 | 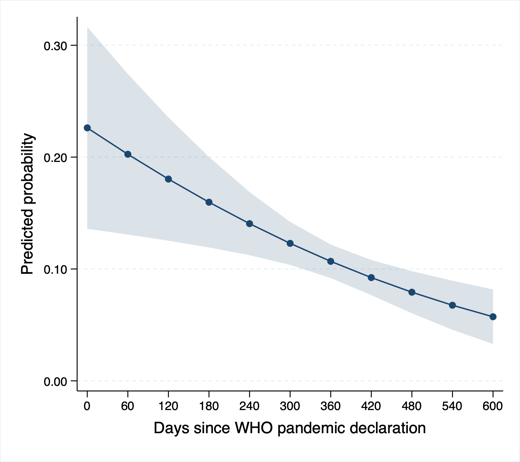 | 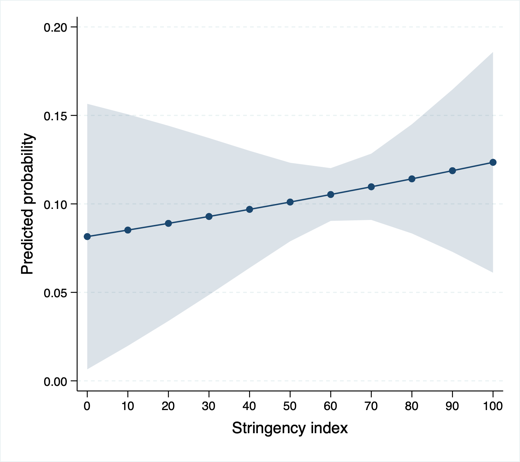 |
|  | | |
| 2) Credit demand | | |
| a) Weeks of temporary closure | b) Days since WHO pandemic declaration | c) Stringency index |
| 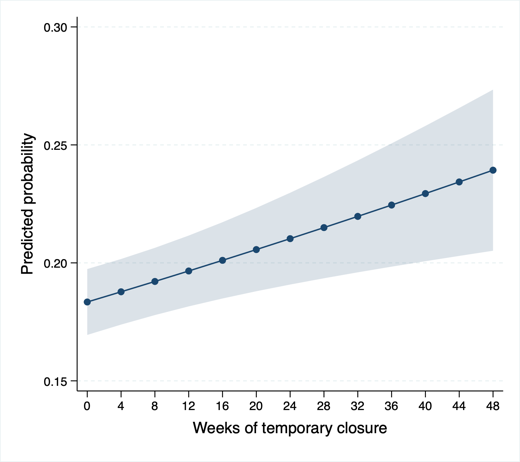 | 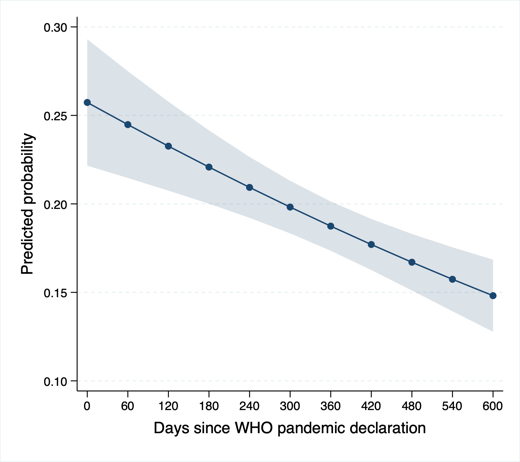 | 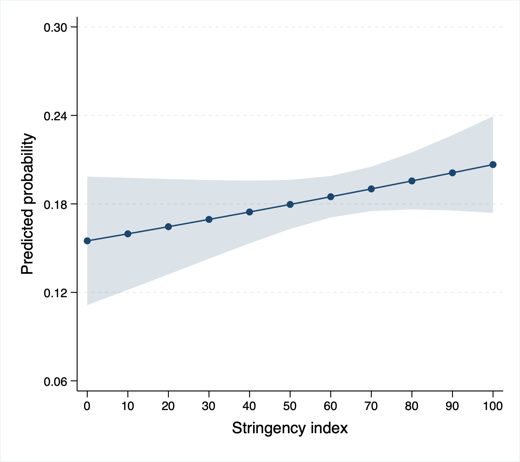 |

Figure S5 – Predicted probabilities of experiencing different pandemic-induced financial issues

Figure S6 – Distribution of the General Management score by country
